# Hydro/Deutero Deamination of Arylazo Sulfones under Metal- and (Photo)Catalyst-Free Conditions

**DOI:** 10.3390/molecules24112164

**Published:** 2019-06-08

**Authors:** Hawraz I. M. Amin, Carlotta Raviola, Ahmed A. Amin, Barbara Mannucci, Stefano Protti, Maurizio Fagnoni

**Affiliations:** 1 PhotoGreen Lab, Department of Chemistry, University of Pavia. Viale Taramelli 12, 27100 Pavia, Italy; hawraz_px@yahoo.com (H.I.M.A.); carlotta.raviola01@universitadipavia.it (C.R.); 2 Chemistry Department, College of Science, Salahaddin University-Erbil, Erbil 44001, Iraq; 3 Chemistry Department, College of Education, Salahaddin University-Erbil, Erbil 44001, Iraq; ahmed.dezaye@su.edu.krd; 4 Centro Grandi Strumenti (CGS), University of Pavia, V. Bassi 21, 27100 Pavia, Italy; barbara.mannucci@unipv.it

**Keywords:** arylazo sulfones, deuteration, visible-light induced processes, photochemistry

## Abstract

Hydrodeaminated and monodeuterated aromatics were obtained via a visible-light driven reaction of arylazo sulfones. Deuteration occurs efficiently in deuterated media such as isopropanol-*d^8^* or in THF-*d^8^*/water mixtures and exhibits a high tolerance to the nature and the position of the aromatic substituents.

## 1. Introduction

Deuterium-labeled compounds, which are physicochemically nearly identical to the non-deuterated analogues, are widely used in mass spectrometry [1] and in NMR spectroscopy [2], as well as in mechanistic elucidation [3,4,5] and metabolic studies [6,7]. Selectively deuterated compounds in medicine are of crucial importance since deuteration was proved to enhance, in some cases, the metabolic stability of drugs [8]. Deuterated drug analogues (also called “heavy drugs”) such as lisofylline (CTP-499) and dextromethorphan (AVP-786) are currently in clinical trials, whereas deuterated tetrabenazine (SD-809) has been recently approved by the FDA for the treatment of chorea associated with Huntington’s disease [9].

**Scheme 1:**
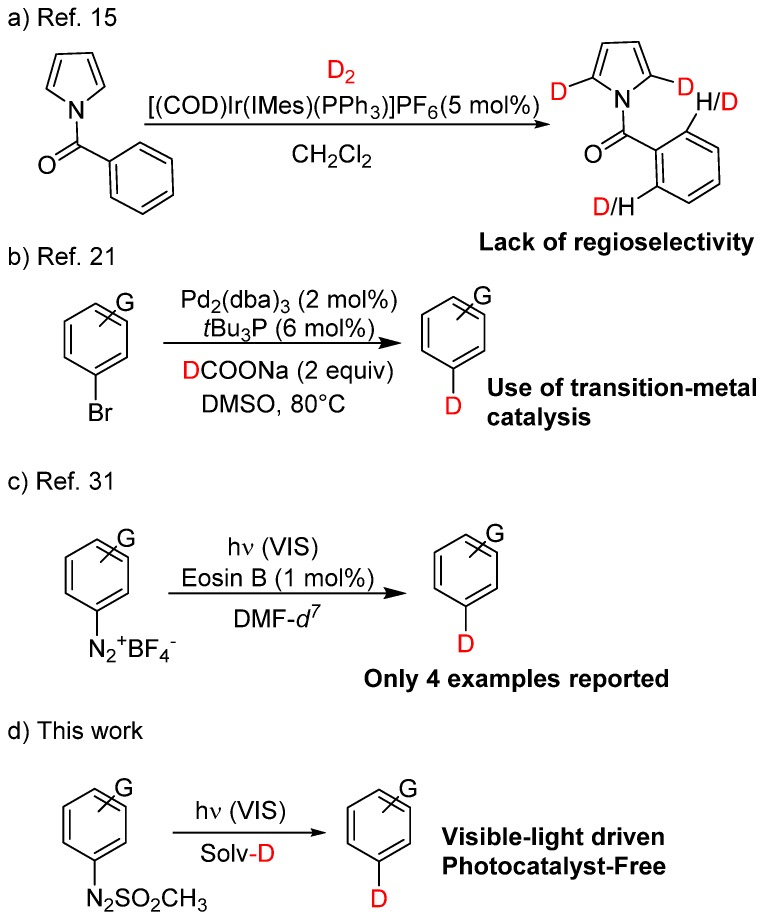
Synthesis of deuterated arenes via (**a**) Ir-catalytic C-H activation, (**b**) Pd-catalyzed deuteration of aryl halides, (**c**) photocatalyzed reductive dediazoniation of arenediazonium salts, (**d**) visible-light induced deuteron deamination of arylazo sulfones.

In view of these premises, the interest for the development of synthetic protocols for the selective deuteration of organic compounds has received much attention in the last decade. Concerning the formation of an Ar–D bond, various processes has been reported based on the activation of an Ar–H [10] or an Ar–X bond. In the former case, the C–H activation of arenes may occur under Pd- [11], Fe- [12], Rh- [13] (in the presence of deuterated acetic or trifluoroacetic acids as the deuterating agents), Ru- [14] and Ir- [15,16] catalysis (D_2_ was used as the deuteron source, see Scheme 1a). Acid/base-mediated labeling strategies making use of D_2_O as a rather inexpensive deuteron source were also proposed [17,18]. However, these transition metal-based approaches often lack regioselectivity [8,9,12], and when using substituted iodoarenes, a dehalogenation and/or alkyl group shift under acid conditions may take place [18]. On the other hand, the Ar–X/Ar–D conversion was achieved via deuterodehalogenation of (hetero)aryl halides (mainly bromides) catalyzed by Pd-complexes [19,20,21] (Scheme 1b), mediated by the potassium methoxide/disilane system [22], by Pd-catalyzed deborylation of boronate esters in THF/D_2_O 4:1 [23] or via deamination of anilines (via in situ prepared diazonium salts) [24].

The use of photochemical and photocatalyzed processes received impressive attention due to the potentiality of visible or solar light as an economic, traceless reagent, and to the mild conditions adopted [25,26,27,28]. In this field, the versatile conversion of aryl halides to the corresponding deuteroarenes, in the presence of porous CdSe nanosheets as the photocatalyst, has been recently described [29], despite the use of Na_2_SO_3_ as the sacrificial electron donor. 

However, photochemistry also offers the chance to carry out chemical processes in the absence of rather expensive and toxic transition metal catalysts/reactants, the presence of which is strictly limited in the preparation of heavy drugs [30]. An interesting example has been reported (for a rather limited range of substrates) and involved the photocatalytic reductive dediazoniation of arenediazonium salts in DMF-*d*^7^ in the presence of Eosin B as the photoorganocatalyst ((POC) [31], Scheme 1c). On the other hand, the adoption of visible-light induced, photocatalyst-free procedures received increasing attention in organic synthesis [32,33,34,35,36].

We recently focused on a class of photoactivable aromatic substrates namely arylazo sulfones. Such derivatives (smoothly obtained in a two-step procedure from the corresponding anilines) bear a dyedauxiliary group (-N_2_SO_2_CH_3_) that, when incorporated in a compound, make it both colored and photoreactive [37]. Indeed, the irradiation with visible light of an arylazo sulfone in polar solvents resulted in the homolytic cleavage of the S–N bond, with the subsequent formation of the corresponding aryl radical. Such behavior has been exploited in the preparation of aromatic amides [38], allylarenes [39], triarylethylenes [40], as well as in the photocatalyst-free, gold catalyzed Suzuki coupling to biaryls [41]. We reasoned that such substrates could be employed in the development of a photocatalyst-free, visible-light driven hydro/deutero deamination process exploiting the well-known hydrogen atom abstraction capability of aryl radicals (Ar^•^, Scheme 1d), as detailed below.

## 2. Results and Discussion

Initial experiments were carried out under deuteron-free conditions on the photoinduced reductive dediazoniation of 1-(methylsulfonyl)-2-(4-acetylphenyl)diazene **1a** to give acetophenone **2**. The reaction was tested in different media, at different concentrations of **1a** and by using different photochemical set-ups. These preliminary results have been summarized in Table S1 (see Electronic Supplementary Information, ESI). The best performance was obtained in the iPrOH/H_2_O 9:1 mixture upon irradiation at 456 nm by means of a Kessil lamp (32 W) under temperature control (25 °C), where **2** was formed in 76% yield. We thus decided to extend such conditions to arylazo sulfones **1b**–**r** (Table 1). The reduction took place for a wide range of substrates and the process exhibited a satisfactory functional group tolerance, since aromatics bearing either electron-withdrawing (e.g., CH_3_CO-, -CN, or -COOMe) or electron-donating (-Me, -*t*Bu, or -OMe) substituents can be efficiently employed. Notably, reduction yield was almost quantitative for halogenated derivatives **1e**–**f** and **1k**, whereas in the case of 2-nitrophenylazo sulfone **1j** the corresponding nitrobenzene **4** was obtained in a discrete yield (41%). The process is still efficient even with polysubstituted aromatics (e.g., **1l** and **1q**). In the case of **1a**, reduction took place efficiently also when increasing the amount of water. A THF/H_2_O 4:1 mixture was used in selected cases as alternative reducing medium, giving comparable results (see the case of **1a** and **1b** in Table 1). 

With these results in our hand we investigated the feasibility of the preparation of deuterated analogues of compounds **2**–**14** (compounds **2-*d^1^*/14-*d^1^***) making use of an isopropanol-*d^8^*/H_2_O 9:1 mixture as the deuteron source. In selected cases, the same reaction was also performed in a THF-*d^8^*/H_2_O 4:1 mixture (Table 2).

Gratifyingly, moving to deuterated solvents did not affect the efficiency of the process and a satisfactory yield of products **2-*d^1^*/14-*d^1^*** was always obtained. In particular, high deuteration yields were found in the case of 4-nitro- and bromo-derivatives **1c**,**e**,**k**, and (poly)methoxyphenylazo sulfone **1q**. Contrary to what observed in non-deuterated media, good results were also obtained when using 2-nitrophenylazo sulfone **1j** since compound **4′*-d^1^*** was formed in 71% yield. α-Deuteronaphthalene **14-*d^1^*** was likewise formed in discrete amounts (64% yield). Noteworthy, the amount of non-deuterated **2**–**14** is always negligible, even in the presence of H_2_O as the co-solvent. This allowed us, in a couple of reactions (the synthesis of **2*-d^1^*** and **6*-d^1^***), to reduce the amount of deuterated isopropanol from 9:1 to 4:1 maintaining comparable yields. The use of a THF*-d_8_*/H_2_O 4:1 mixture as the medium often led to a lower efficiency of the process (compare for instance the yields obtained for **7*-d^1^*** and **12*-d^1^***). The deuteration reaction is essentially clean (see Table 2 and ESI). 

We carried out preparative reactions (on a 0.1 mmol scale) only in selected cases due to the volatility of the other aromatics. Thus, deuterated arenes **4-*d^1^***, **13-*d^1^*** and **14-*d^1^*** were isolated in satisfactory yields (54–89%, see Table 2), pointing out the synthetic potentiality of our approach. 

**Scheme 2:**
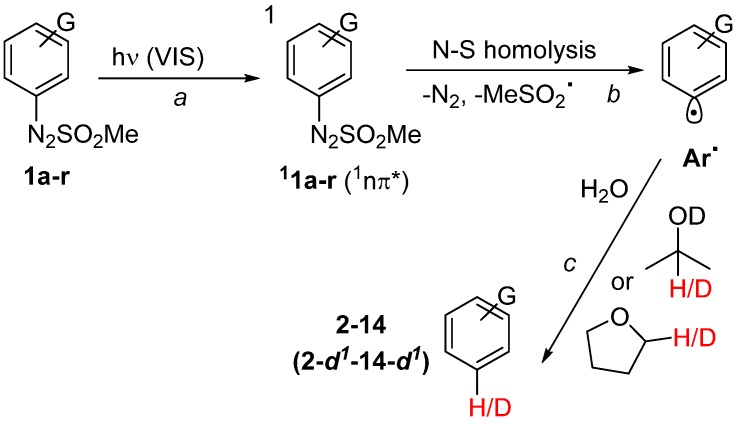
Plausible mechanism for the visible light induced dediazoniation of arylazo sulfones **1a**–**r** to form hydrogenated/deuterated derivatives **2**–**14/2-*d^1^***–**14-*d^1^***.

Based on the observed results, we suggested the mechanism described in Scheme 2. Arylazo sulfones (**1a**–**r**) show a wavelength-selective behavior [37,42] where the homolytic cleavage of the N–S bond and the subsequent formation of an aryl radical (**Ar^•^**, path b) takes place from the singlet excited state (^1^nπ*) upon visible light irradiation (path a).

Hydrogen atom transfer (HAT) between **Ar^•^** and the surrounding environment (path c) is efficient and widely investigated in the literature [43,44,45,46,47,48]. Our results confirmed that such highly reactive (but yet selective) **Ar^•^** selectively abstracts a hydrogen (or a deuteron) from a C–H bond (or a C–D bond) in both isopropanol (C–H BDE = 91 ± 1.0 kcal mol^−1^) and THF (C–H BDE = 92.1 ± 1.6 kcal mol^−1^ [35]; the C–H/C–D bond cleaved is the weakest one as indicated in red in Scheme 2) to form the corresponding Ar–H or Ar–D product (Ar–H BDE in benzene: 112.9 ± 0.5 kcal mol^−1^ [49]). The presence of a significant amount of non-deuterated water (up to ca. 11 M in the case of a deuterated isopropanol/H_2_O 4:1 mixture) did not appreciably affect the deuteration yield.

## 3. Conclusions

Summing up, with the “proof of concept” presented herein we highlighted the potentialities of bench stable, colored arylazo sulfones in the preparation of deuterated aromatics via visible light irradiation at room temperature under both metal- and photocatalyst-free conditions.

## 4. Materials and Methods

### 4.1. General

^1^H- and ^13^C-NMR spectra were recorded on a 300 MHz spectrometer (Bruker, Milan, Italy), chemical shifts were reported in ppm downfield from TMS, and the attributions were made based on ^1^H and ^13^C signals; chemical shifts were reported in ppm downfield from TMS.

The reaction course was followed by means of GC-MS. GC-MS analyses were carried out using a Thermo Scientific DSQII single quadrupole GC/MS system (Thermo Scientific®, San Jose, CA, USA).

A Restek Rtx-5MS (30 m × 0.25 mm × 0.25 μm) capillary column (Restek Corporation, Bellefonte, USA) was used for analyte separation with helium as carrier gas at 1 mL min^−1^. The injection in the GC system was performed in split mode and the injector temperature was 250 °C. The GC oven temperature was held at 80 °C for 2 min, increased to 220 °C by a temperature ramp of 10 °C min^−1^ and held for 10 min. The transfer line temperature was 250 °C and the ion source temperature 250 °C. Mass spectral analyses were carried out in full scan mode. Deuterated solvents were commercially available and were used as received. Arylazo sulfones (**1a**–**1e**, **1g**, **1i**, **1j**, **1n**–**p**, and **1r,** [38]; **1f** [50]; and **1m** [40]) were previously synthesized and fully characterized in our lab.

### 4.2. General Procedure for the Synthesis of Arylazo Sulfones (***1h**, **1k**, **1l**, **1q***)

Arylazo sulfones (**1h**, **1k**, **1l**, and **1q**) were synthesized according to the literature procedure [37]. Diazonium salts were freshly prepared prior to use from the corresponding anilines and purified by dissolving in acetonitrile and precipitation by adding cold diethyl ether. To a cooled (0 °C) suspension of the chosen diazonium salt (1 equiv, 0.3 M) in CH_2_Cl_2_ was added sodium methanesulfinate (1.2 equiv) in one portion. The temperature was allowed to rise to room temperature, and the solution stirred overnight. The resulting mixture was then filtered, and the obtained solution was evaporated. The crude product was finally dissolved in CH_2_Cl_2_ and precipitated by adding cold *N*-hexane.

*1-(Methylsulfonyl)-2-(3-acetylphenyl)diazene* (**1h**). From 3-acetylphenydiazonium tetrafluoroborate [51] (1.50 g, 6.4 mmol) and 790 mg (1.2 equiv) of sodium methanesulfinate in CH_2_Cl_2_ (21 mL). Recrystallization afforded 999 mg of 1-(methylsulfonyl)-2-(4-acetylphenyl)diazene (**1h**, yellow solid, 69% yield, mp: 74–76 °C dec). ^1^H-NMR (300 MHz, CD_3_COCD_3_, δ): 2.73 (s, 3H), 3.35 (s, 3H), 7.84-7.89 (t, 1H, *J* = 7.5 Hz), 8.19–8.22 (dd, 1H, *J* = 7 and 2 Hz), 8.35–8.39 (dd, 1H, *J* = 7 and 2 Hz), 8.52–8.54 (d, 1H, *J* = 2.5 Hz). ^13^C-NMR (75 MHz, CD_3_COCD_3_, δ): 27.3 (CH_3_), 35.4 (CH_3_), 125.25 (CH), 128.4 (CH), 131.7 (CH), 135.3 (CH), 140.2, 150.7, 197.3. IR (neat, ν/cm^−1^): 3056, 2992, 1690, 1340, 1146.

*1-(Methylsulfonyl)-2-(2-bromophenyl)diazene* (**1k**). From 2-bromophenydiazonium tetrafluoroborate [52] (1.50 g, 5.5 mmol) and 680 mg (1.2 equiv) of sodium methanesulfinate in CH_2_Cl_2_ (21 mL). Recrystallization afforded 706 mg of 1-(methylsulfonyl)-2-(2-bromophenyl)diazene (**1k**, yellow solid, 49% yield, mp: 97.6–99.3 °C dec). ^1^H-NMR (300 MHz, CD_3_COCD_3_, δ): 3.35 (s, 3H), 7.64–7.78 (m, 3H) 7.98–8.01 (dd, 1H, *J* = 8 and 1.5 Hz). ^13^C-NMR (75 MHz, CD_3_COCD_3_, δ): 35.6 (CH_3_), 119.3 (CH), 128.8, 130.3 (CH), 136.0 (CH), 137.7 (CH), 147.7. IR (neat, ν/cm^−1^): 3056, 2992, 1690, 1340, 1146. IR (neat, ν/cm^−1^): 3060, 2990, 1342, 1156.

*1-(Methylsulfonyl)-2-(2-chloro-4-fluorophenyl)diazene* (**1l**). From 2-chloro-4-fluorophenydiazonium tetrafluoroborate [53] (1.50 g, 6.1 mmol) and 753 mg (1.2 equiv) of sodium methanesulfinate in CH_2_Cl_2_ (21 mL). Recrystallization afforded 910 mg of 1-(methylsulfonyl)-2-(2-chloro-4-fluorophenyl)diazene (**1l**, yellow solid, 63% yield, mp: 73–75 °C dec). ^1^H-NMR (300 MHz, CD_3_COCD_3_, δ): 3.35 (s, 3H), 7.39–7.46 (m, 1H), 7.68–7.71 (dd, 1H, *J* = 8 and 2.5 Hz), 7.91–8.00 (m, 1H). ^13^C-NMR (75 MHz, CD_3_COCD_3_, δ): 35.6 (CH_3_), 117.4 (d, CH, *J* = 23 Hz), 119.9 (d, CH, *J* = 26 Hz), 121.4 (d, CH, *J* = 10.5 Hz), 140.7 (d, J = 11 Hz), 143.5 (d, *J* = 3 Hz), 167.7 (d, J = 258 Hz). IR (neat, ν/cm^−1^): 3099, 3037, 2937, 1587, 1345, 1161.

*1-(Methylsulfonyl)-2-(3,4,5-trimethoxyphenyl)diazene* (**1q**). From 3,4,5-trimethoxybenzenediazonium tetrafluoroborate [54] (1.50 g, 5.3 mmol) and 654 mg (1.2 equiv) of sodium methanesulfinate in CH_2_Cl_2_ (21 mL). Recrystallization afforded 654 mg of 1-(methylsulfonyl)-2-(3,4,5-trimethoxyphenyl)diazene (**1i**, yellow solid, 45% yield, mp: 114–116 °C dec). ^1^H-NMR (300 MHz, CD_3_COCD_3_, δ): 2.29 (s, 3H), 3.91 (s, 3H), 3.97 (s, 6H), 7.35 (s, 2H). ^13^C-NMR (75 MHz, CD_3_COCD_3_, δ): 35.3 (CH_3_), 57.1 (CH_3_), 61.4 (CH_3_), 103.5 (CH), 105.9, 146.0, 155.3. IR (neat, ν/cm^−1^): 3055, 2941, 1471, 1343, 1129.

### 4.3. General Procedure for Photochemical Irradiations

A 0.025 M solution of **1a**–**r** in the chosen medium (*i*PrOH-H_2_O 9:1, THF-H_2_O 4:1, isopropanol-*d*^8^/H_2_O 9:1, isopropanol-*d*^8^/H_2_O 4:1, and THF-*d^8^*/H_2_O 4:1, 1 mL) was irradiated at 456 nm (Kessil lamp, 32W) for 14 h. An air-cooling system was used to maintain the temperature below 30 °C. The reaction course and the product distribution were analyzed by GC-MS analyses. The amounts of compounds **2**–**14/2-*d^1^*-14-*d^1^*** have been determined by using calibration curves.

In selected cases, deuteration of arylazo sulfones was scaled-up and carried out on a 0.1 mmol scale.

Synthesis of 4-deutero-1-nitrobenzene (**4*-d^1^***): A 0.1 M solution of **1d** (22.4 mg, 0.1 mmol) in an isopropanol-*d*^8^/H_2_O 9:1 mixture (1 mL) was irradiated for 24 h at 456 nm, then the solvent evaporated in vacuo. Purification of the resulting residue by column chromatography (eluant: Neat pentane) afforded 8.5 mg of **4-*d^1^*** (oil, 68% yield). The spectroscopic data of **4-*d^1^*** were in accordance with the literature [19].

Synthesis of 4-deutero-1,2,3-trimethoxybenzene (**13-*d^1^***): A 0.1 M solution of **1q** (27.4 mg. 0.1 mmol) in isopropanol-*d*^8^/H_2_O 9:1 mixture (1 mL) was irradiated for 24 h at 456 nm, then the solvent evaporated in vacuo. Purification of the resulting residue by column chromatography (eluant: Neat cyclohexane) afforded 15 mg of **13-*d^1^*** (pale yellow solid, mp = 38–40 °C, 89% yield). The spectroscopic data of **13-*d^1^*** were in accordance with the literature [55].

**13-*d^1^***. ^1^H-NMR (300 MHz, CDCl_3_, δ): 3.88 (s, 3H), 3.89 (s, 6H), 6.61 (s, 2H). ^13^C-NMR (75 MHz, CDCl_3_, δ): 56.0 (CH_3_), 66.7 (CH_3_), 105.0 (CH), 123.2 (t, *J* = 20 Hz, CD), 138.0, 153.4.

Synthesis of α-deutero-naphthalene (**14-*d^1^***): A 0.1 M solution of **1r** (23.4 mg, 0.1 mmol) in an isopropanol-*d^8^*/H_2_O 9:1 mixture (1 mL) was irradiated for 24 h at 456 nm, then the solvent evaporated under vacuo. Purification of the resulting residue by column chromatography (eluant: Neat pentane) afforded 7 mg of **14-*d^1^*** (pale yellow solid, mp = 70.2–71.3 °C, lit. 71–73 °C [56], 54% yield). The spectroscopic data of **14-*d^1^*** were in accordance with the literature [55].

## Figures and Tables

**Table 1:** Reductive dediazoniation of arylazo sulfones. Substrate scope ^a^.

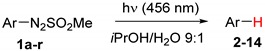	
**Ar-N_2_SO_2_Me**	**Product, % Yield**
**1a**, Ar = 4-CH_3_CO-C_6_H_4_	**2**, 76, 71 ^b^, 61 ^c^
**1b**, Ar = 4-CN-C_6_H_4_	**3**, 68, 54 ^c^
**1c**, Ar = 4-NO_2_-C_6_H_4_	**4**, 55
**1d**, Ar = 4-Cl-C_6_H_4_	**5**, 82
**1e**, Ar = 4-Br-C_6_H_4_	**6**, 97
**1f**, Ar = 4-I-C_6_H_4_	**7**, 97
**1g**, Ar = 4-COOMe-C_6_H_4_	**8**, 79
**1h**, Ar = 3-CH_3_CO-C_6_H_4_	**2**, 77
**1i**, Ar = 3-CN-C_6_H_4_	**3**, 54
**1j**, Ar = 2-NO_2_-C_6_H_4_	**4**, 41
**1k**, Ar = 2-Br-C_6_H_4_	**6**, 97
**1l**, Ar = 2-Cl, 4-F-C_6_H_3_	**9**, 57
**1m**, Ar = 4-Me-C_6_H_4_	**10**, 75
**1n**, Ar = 4-*t*Bu-C_6_H_4_	**11,** 78, 55 ^c^
**1o**, Ar = 4-CH_3_O-C_6_H_4_	**12**, 55
**1p**, Ar = 2-CH_3_O-C_6_H_4_	**12**, 80
**1q**, Ar = 3,4,5-CH_3_O-C_6_H_2_	**13**, 89
**1r**, Ar = α-Naphthyl	**14**, 57

^a^ Reaction conditions. A solution of the chosen arylazo sulfone **1a–r** (0.025 M) in an *i*PrOH/H_2_O 9:1 mixture irradiated for 14 h at 456 nm (32 W Kessil lamp). ^b^ Irradiation carried out in an *i*PrOH/H_2_O 4:1 mixture. ^c^ Irradiation carried out in a THF/H_2_O 4:1 mixture.

**Table 2:**
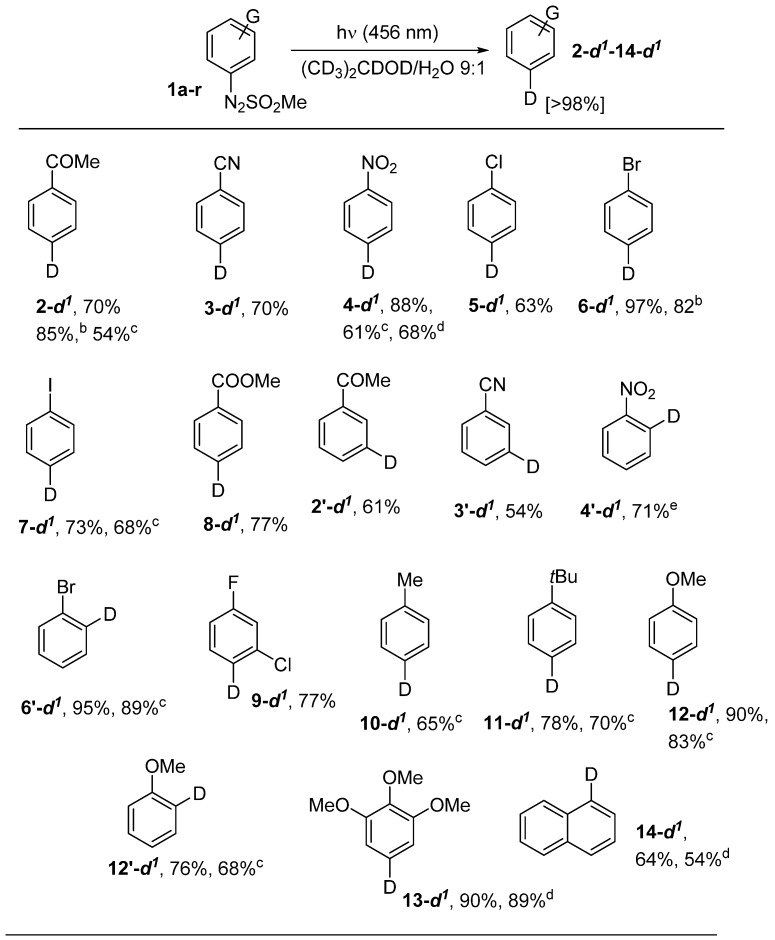
Preparation of deuterated arenes (**2-*d^1^*-14-*d^1^***) ^a^.

^a^ Reaction conditions: **1a**–**r** (0.025 M) in an isopropanol-*d*^7^/H_2_O 9:1 mixture irradiated at 456 nm (Kessil lamp, 32W) for 14 h. ^b^ Reaction carried out in an isopropanol-*d*^7^/H_2_O 4:1 mixture. ^c^ Reaction carried out in a THF-*d^8^*/H_2_O 4:1 mixture. ^d^ Reaction carried out in an isopropanol-*d*^7^/H_2_O 9:1 mixture on a 0.1 mmol scale (0.1 M, 1 mL), isolated yield. ^e^ 2-Nitrophenyl methylsulfone (<10%) detected by GC-MS.

## Supplementary Materials

The following are available online, MS spectra of compounds **2-*d^1^*-14-*d^1^***; ^1^H- and ^13^C-NMR spectra of **1h**, **1k**, **1l**, **1q**, **4-*d^1^***, **13-*d^1^***, and **14-*d^1^*** [56,57,58,59,60,61,62,63,64,65].
