# Supplementary material for: Hydro/Deutero Deamination of Arylazo Sulfones under Metal- and (Photo)Catalyst-Free Conditions
Source: Molecules. 2019 Jun 8;24(11):2164. doi: 10.3390/molecules24112164 (PMC6601019; doi:10.3390/molecules24112164)
Supplement: Supplementary file 1 [file molecules-24-02164-s001.pdf]

Supporting Information

Hydro/Deutero Deamination of Arylazo Sulfones under Metal- and (Photo)Catalyst-Free Conditions

Hawraz I. M. Amin [1,2], Carlotta Raviola [1], Ahmed A. Amin [3], Barbara Mannucci [4], Stefano Protti [1,*] and Maurizio Fagnoni [1,*]

[1] PhotoGreen Lab, Department of Chemistry, University of Pavia. Viale Taramelli 12, 27100 Pavia, Italy; hawraz_px@yahoo.com (H.I.M.A.); carlotta.raviola01@universitadipavia.it (C.R.)

[2] Chemistry Department, College of Science, Salahaddin University-Erbil, Erbil 44001, Iraq

[3] Chemistry Department, College of Education, Salahaddin University-Erbil, Erbil 44001, Iraq; ahmed.dezaye@su.edu.krd

[4] Centro Grandi Strumenti (CGS), University of Pavia, V. Bassi 21, 27100 Pavia, Italy; barbara.mannucci@unipv.it

* Correspondence: stefano.protti@unipv.it (S.P.); fagnoni@unipv.it (M.F.); Tel.: +390382987314

Table of contents

1. Optimization of the procedure for the reductive dediazoniation of arylazo sulfones **S3**

2. MS spectra of compounds 2-*d*^{1}-14-*d*^{1} and ^{1}H and ^{13}C NMR of 13-*d*^{1} **S5**

3. ^{1}H and ^{13}C NMR spectra of compounds 1h, 1k, 1l, 1q **S26**

4. References **S34**

1 Optimization of the procedure for the reductive dediazoniation of arylazo sulfones.

Table S1. Irradiation of arylazosulfone **1a** in different solvents.[a]

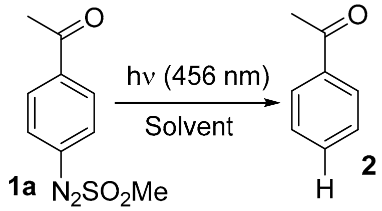

Entry	Conditions	2 (% yield)
1	**1a** (0.025 M), MeOH	48
2	**1a** (0.025 M), MeOH-H_2O 9:1	50
3	**1a** (0.025 M), MeOH-H_2O 4:1	51
4	**1a** (0.025 M), MeCN	30
5	**1a** (0.025 M), MeCN-H_2O 9:1	38
6	**1a** (0.025 M), MeCN-H_2O 4:1	43
7	**1a** (0.025 M), Acetone	54[b]
8	**1a** (0.05 M), Acetone	49[b]
9	**1a** (0.1 M), Acetone	34[b]
10	**1a** (0.025 M), THF	59
11	**1a** (0.025 M), THF-H_2O 9:1	56
12	**1a** (0.025 M), THF-H_2O 4:1	61
**13**	**1a (0.025 M), *i*PrOH-H_2O 9:1**	**76**
14	**1a** (0.025 M), *i*PrOH-H_2O 4:1	71
15[c]	**1a** (0.025 M), *i*PrOH-H_2O 9:1	[c]

[a] A solution of **1a** in the chosen medium (1 mL) was nitrogen purged for 5 min, then irradiated by means of a 34 W Kessil Lamp (λ_{em} = 456 nm). The reaction course was monitored by GC analyses and the amount of **2** quantified by a calibration curve. [b] 4-Acetylphenyl methylsulfone (<10%) detected by GC-MS [c] Blank experiment, no consumption of **1a** observed.

As depicted in Table S1, irradiation of a 0.025 M solution of **1a** in methanol as well as in different methanol-water mixtures (entries 1-3) afforded a moderate yield of acetophenone **2**. A similar behavior was observed in acetonitrile containing mixtures (entries 4-6), while when moving to acetone, a noxious effect on the yield of **2** was observed when increasing the concentration of **1a** (entries 7-9). Furthermore, small amounts (<10% yield) of the by-product 4-acetylphenyl methyl sulfone (GC-MS: 198 (6), 183 (100), 121 (54))[S1] were detected. Better yields were obtained by choosing THF as the (co)solvent (up to 61% yield, entries 10-12). However, when shifting to aqueous isopropanol (entries 13,14), conversion of **1a** to **2** was found to occur in a satisfactory yield (76% in

*i*PrOH-H_2O 9:1 mixture). Finally, no consumption of **1a** took place in the absence of irradiation (entry 15). The protocol described in entry 13 was thus adopted to investigate the versatility of the reaction.

2. MS spectra of compounds 2-d^1-14-d^1 and ^{1}H and ^{13}C NMR of 13-d^1

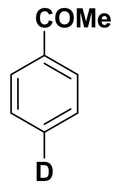

2-d^1

Reference: S2

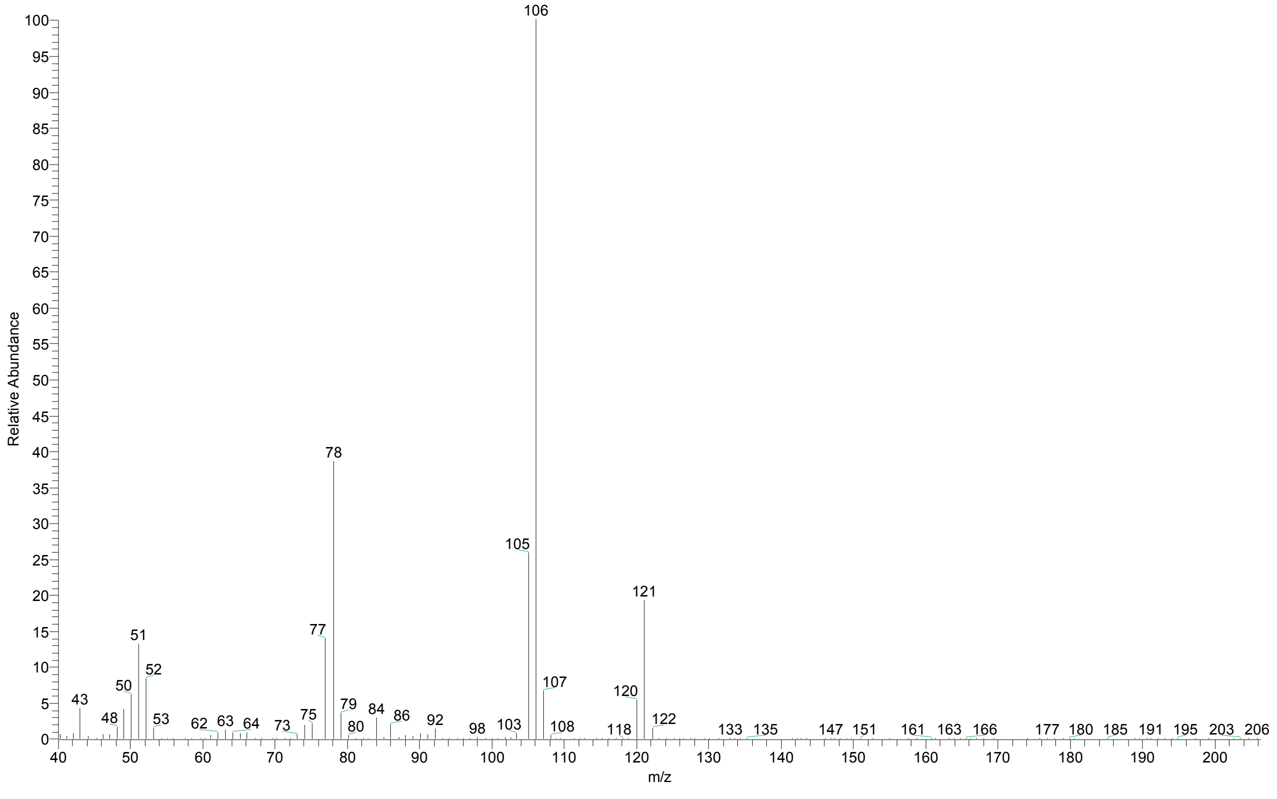

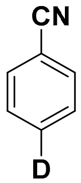

3-*d*[1]

Reference: S3

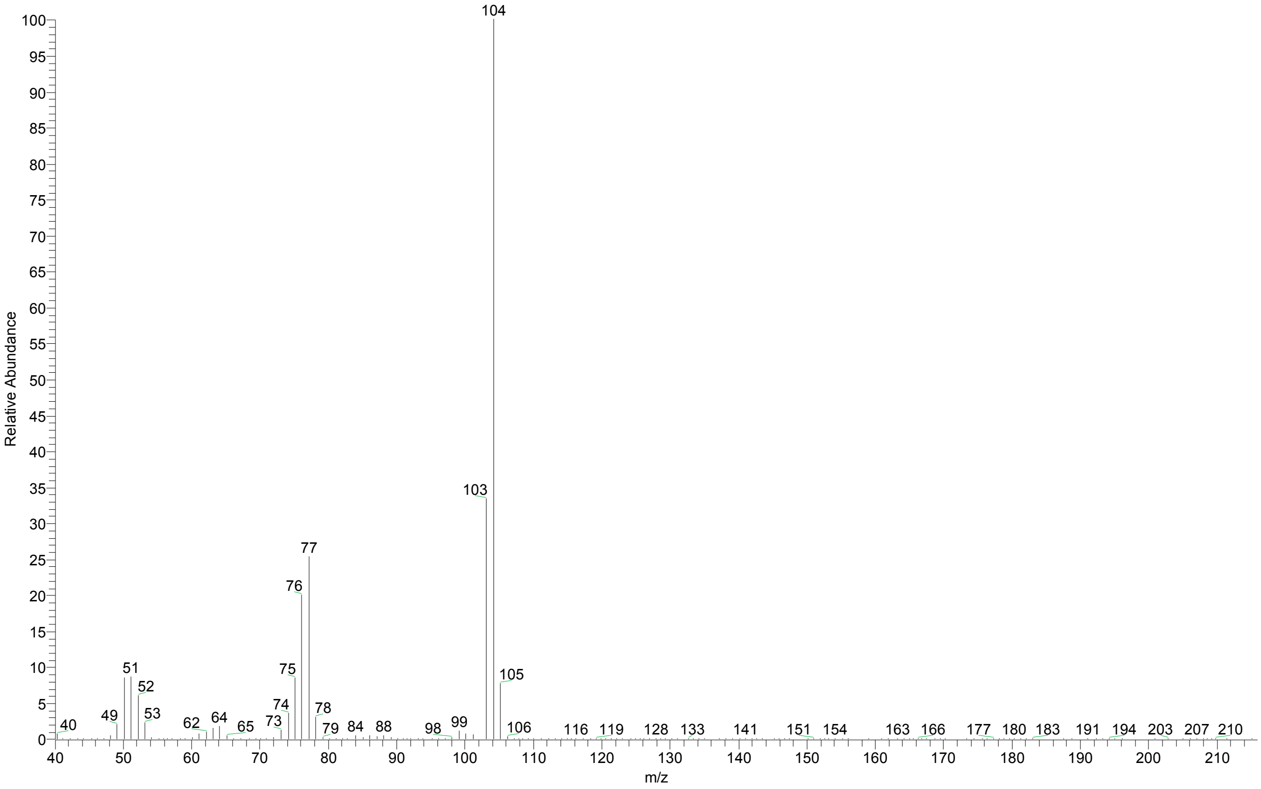

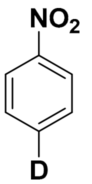

4-*d*[1]

Reference: S3

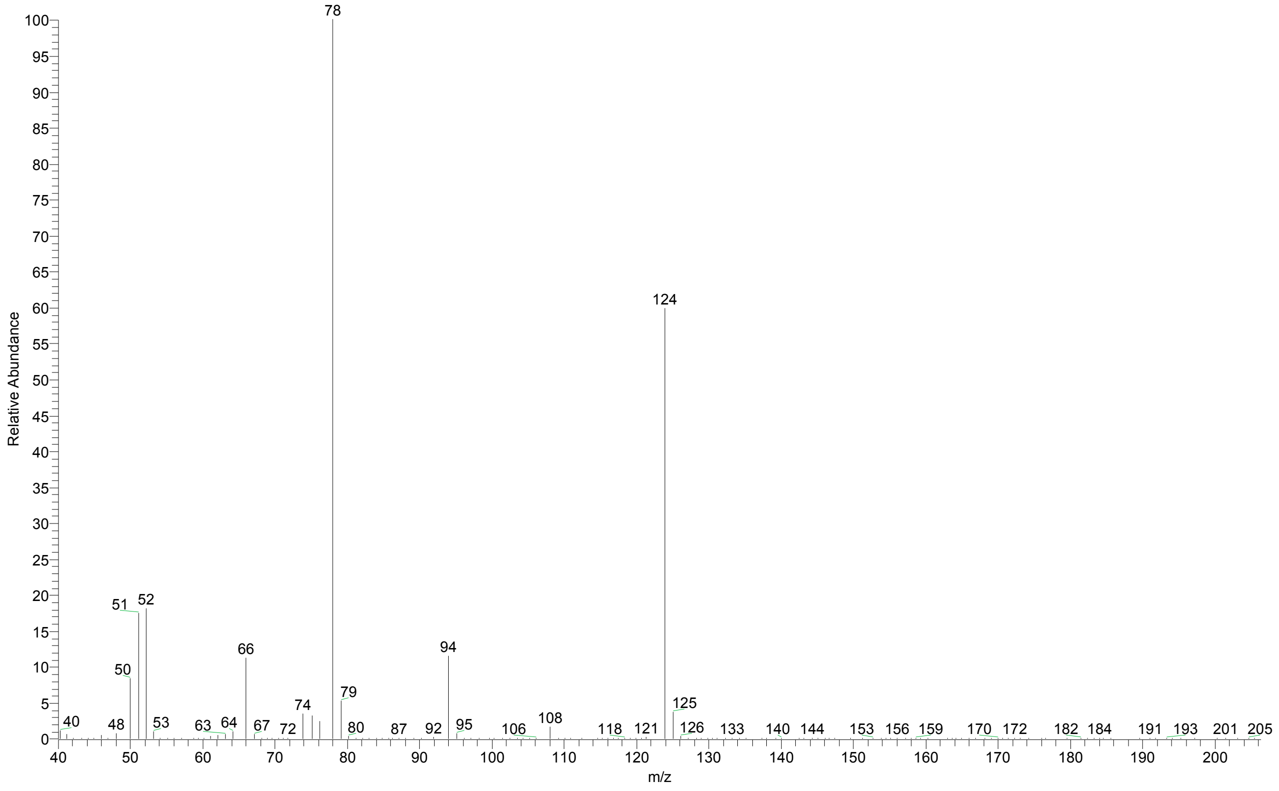

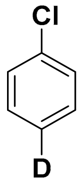

5-*d*[1]

Reference: S4

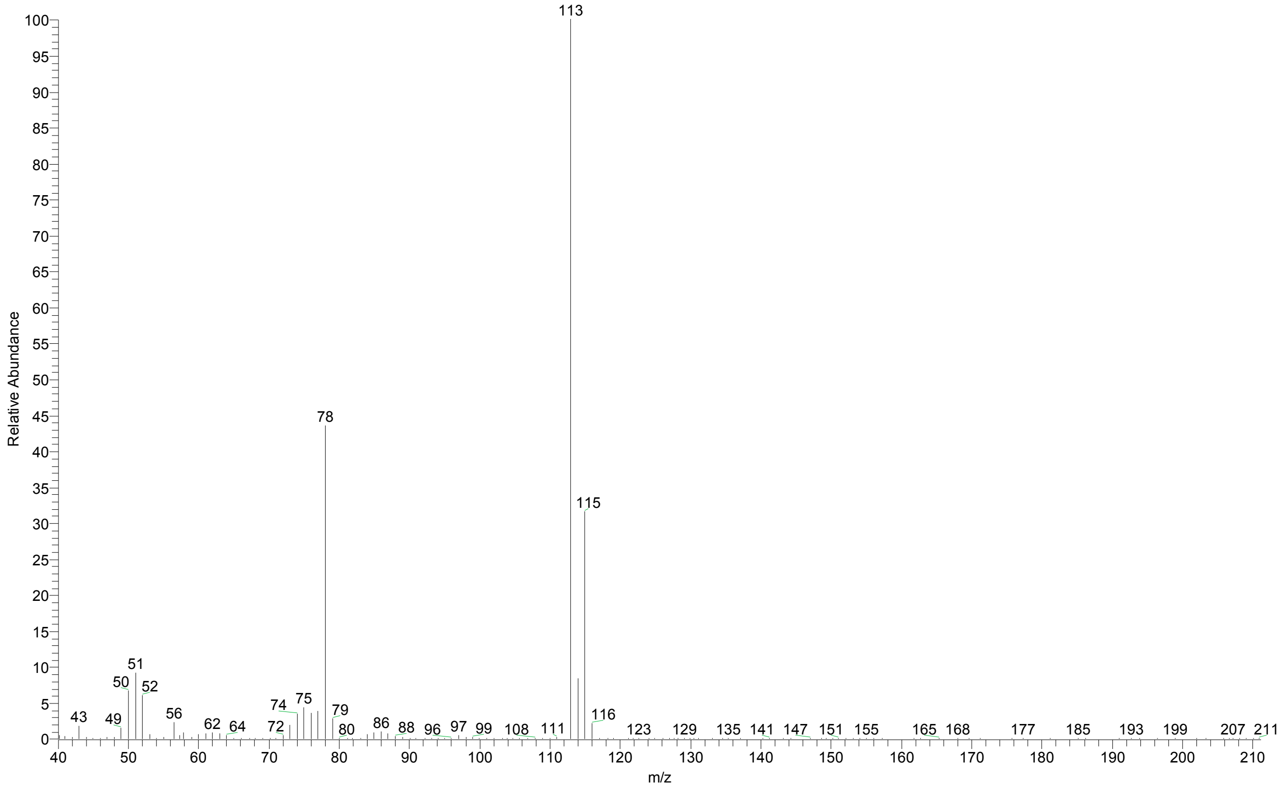

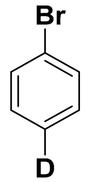

6-*d*[1]

Reference: S5

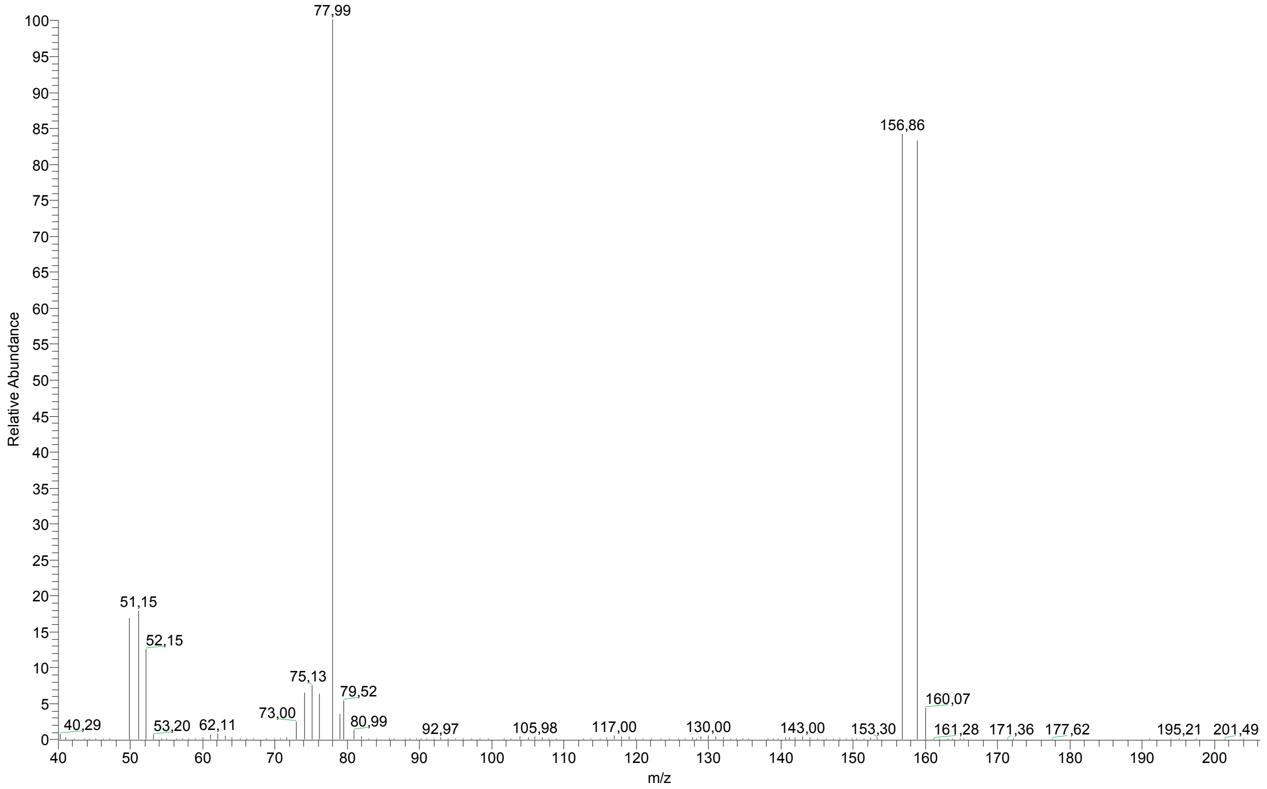

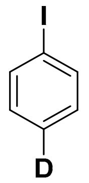

***7-d*[1]**

Reference: S6

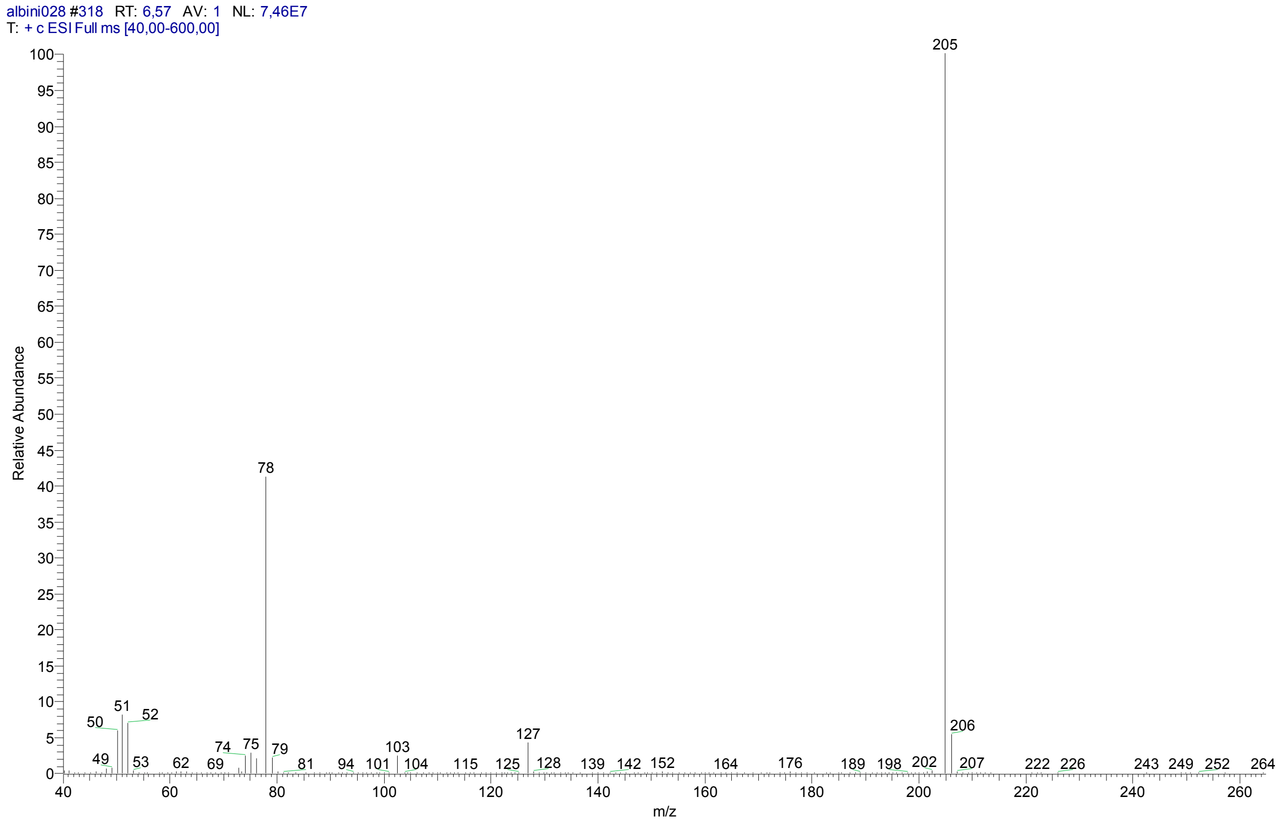

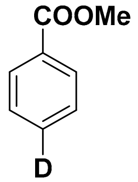

8-*d*[1]

Reference S7

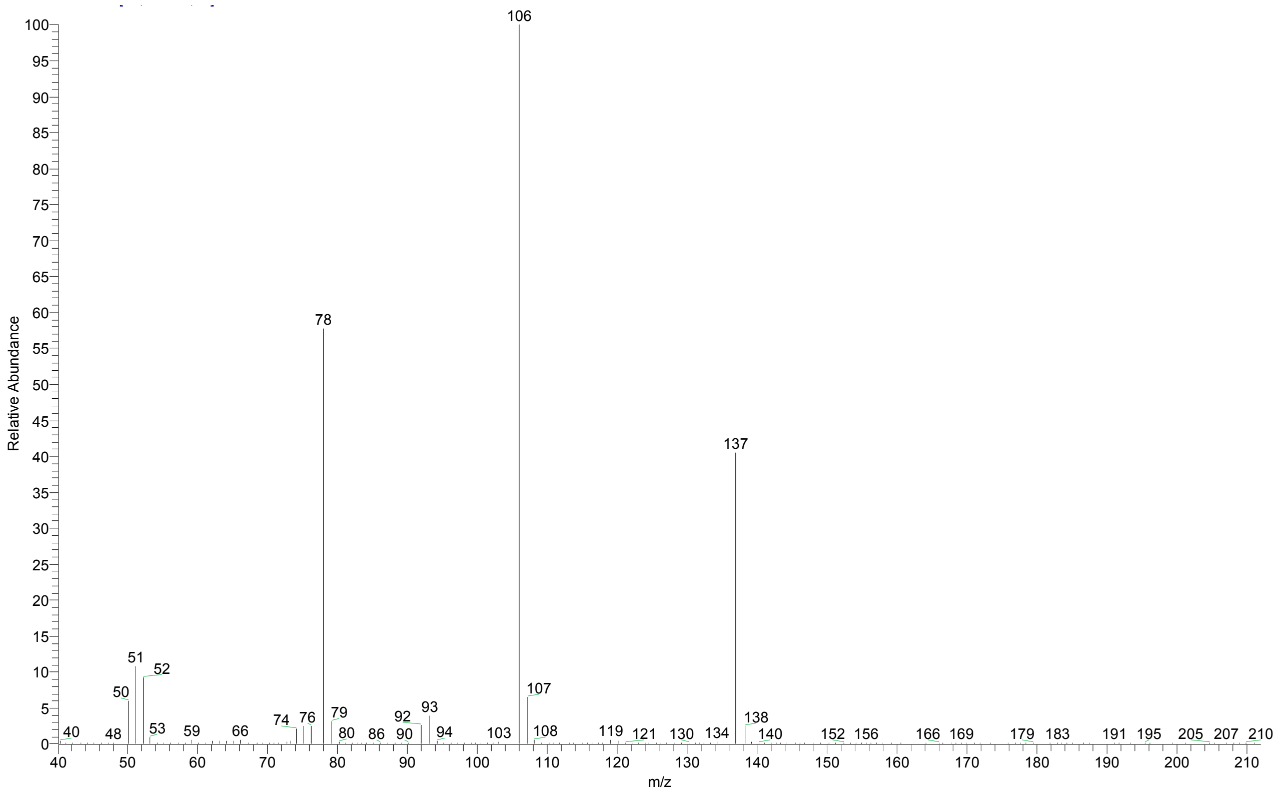

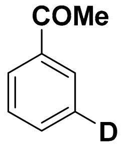

***2'-d*^{1}**

Reference: S8

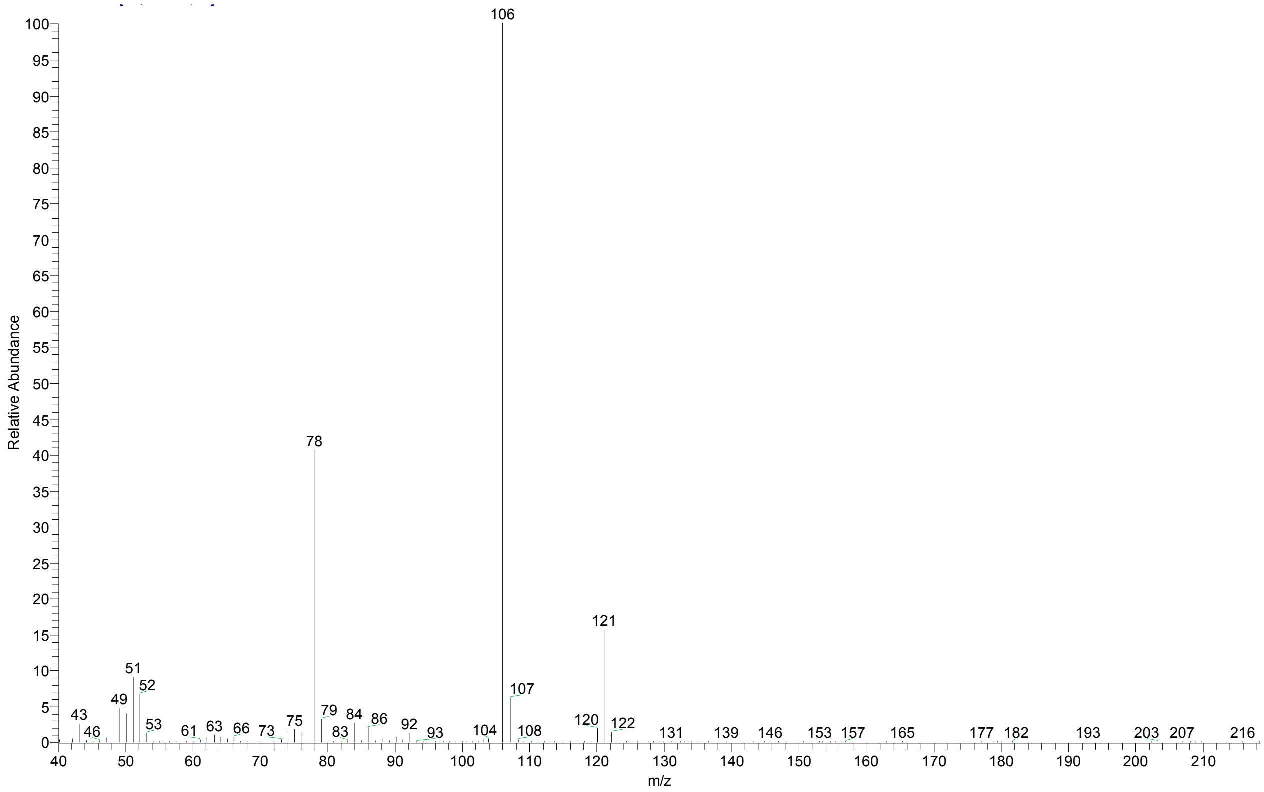

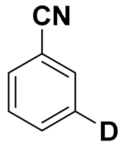

3'-d[1]

Reference: S9

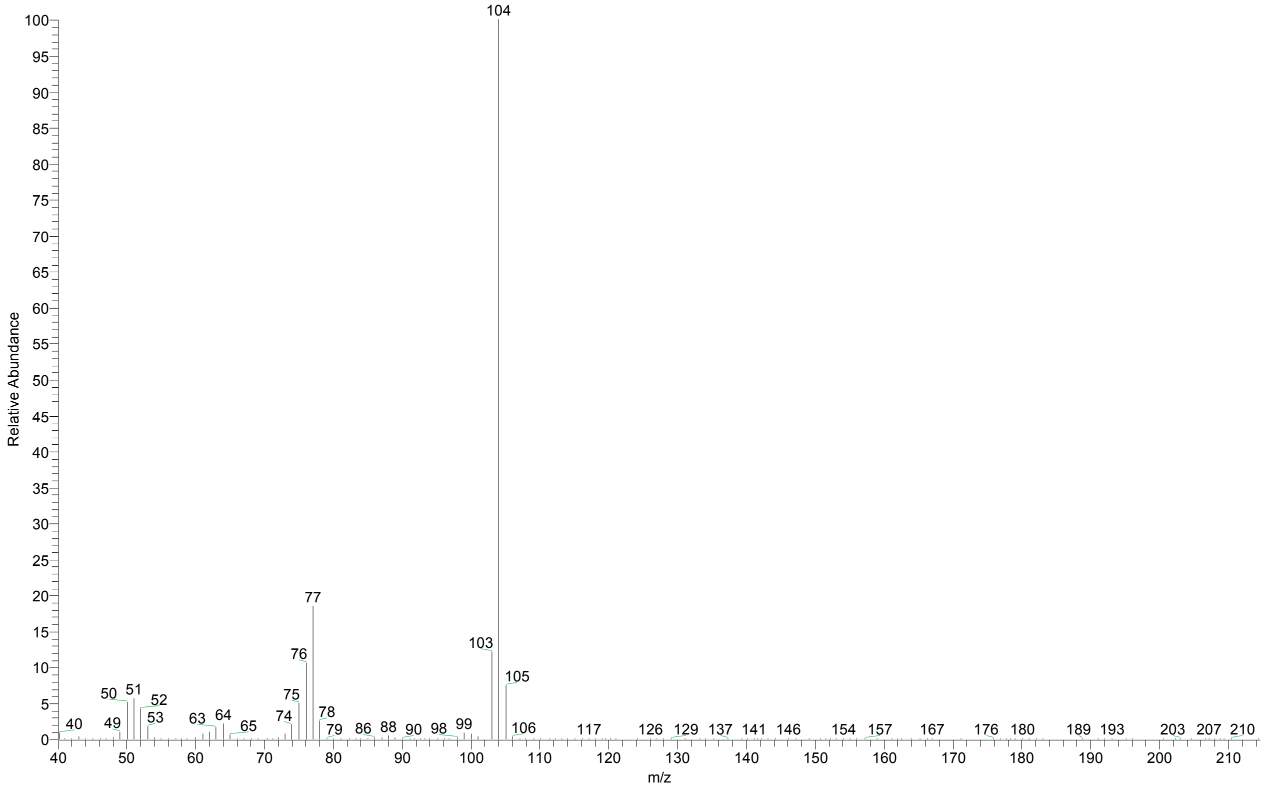

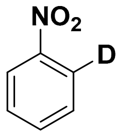

***4'-d*[1]**

Reference: S10

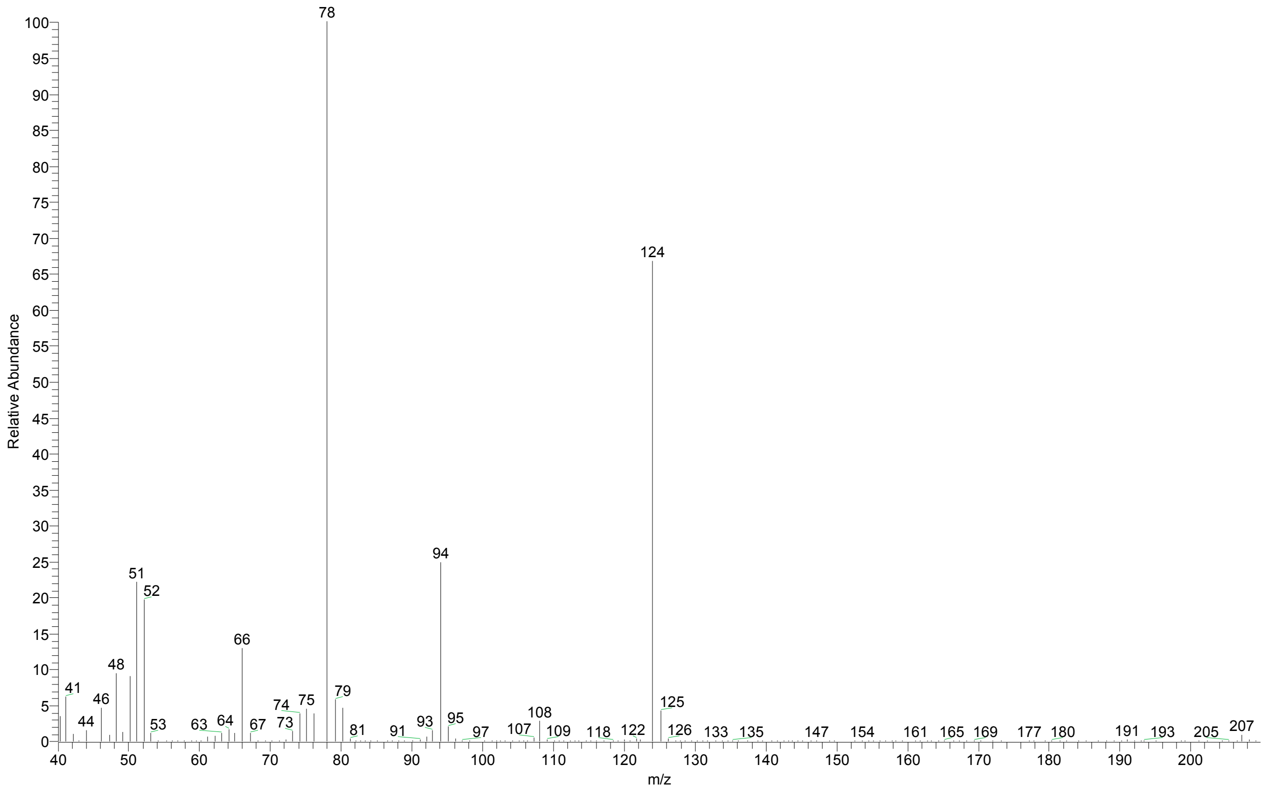

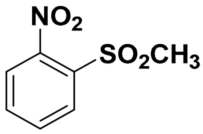

Reference: S11

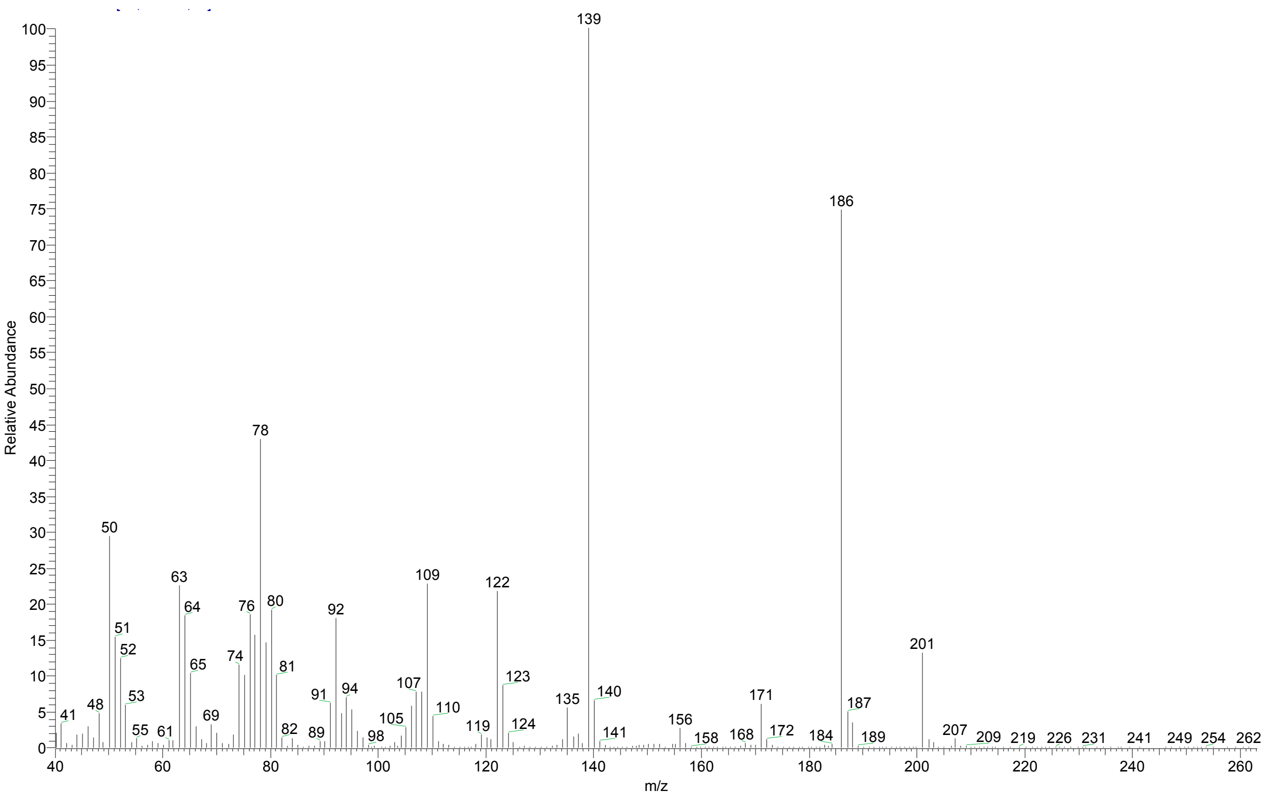

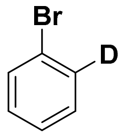

***6'-d*^1**

Reference: S12

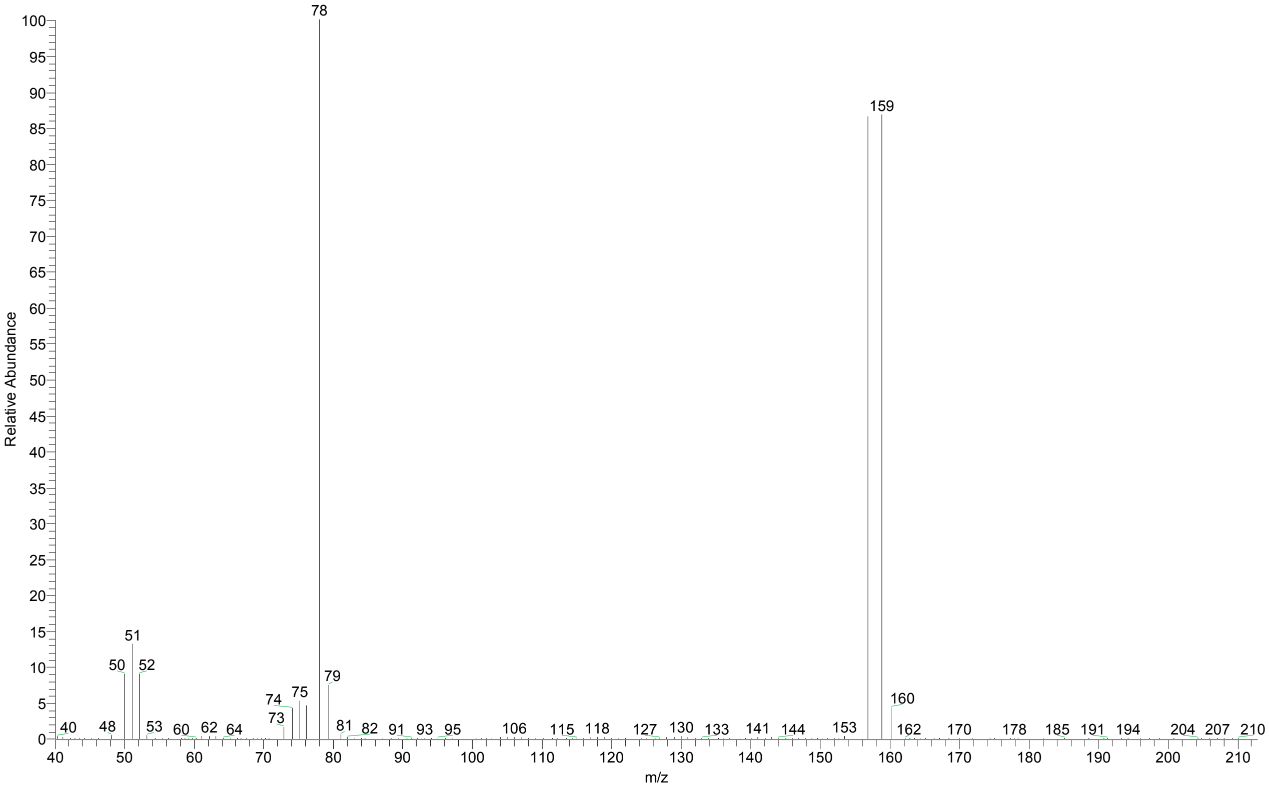

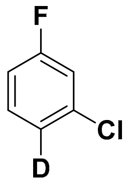

9-d^1

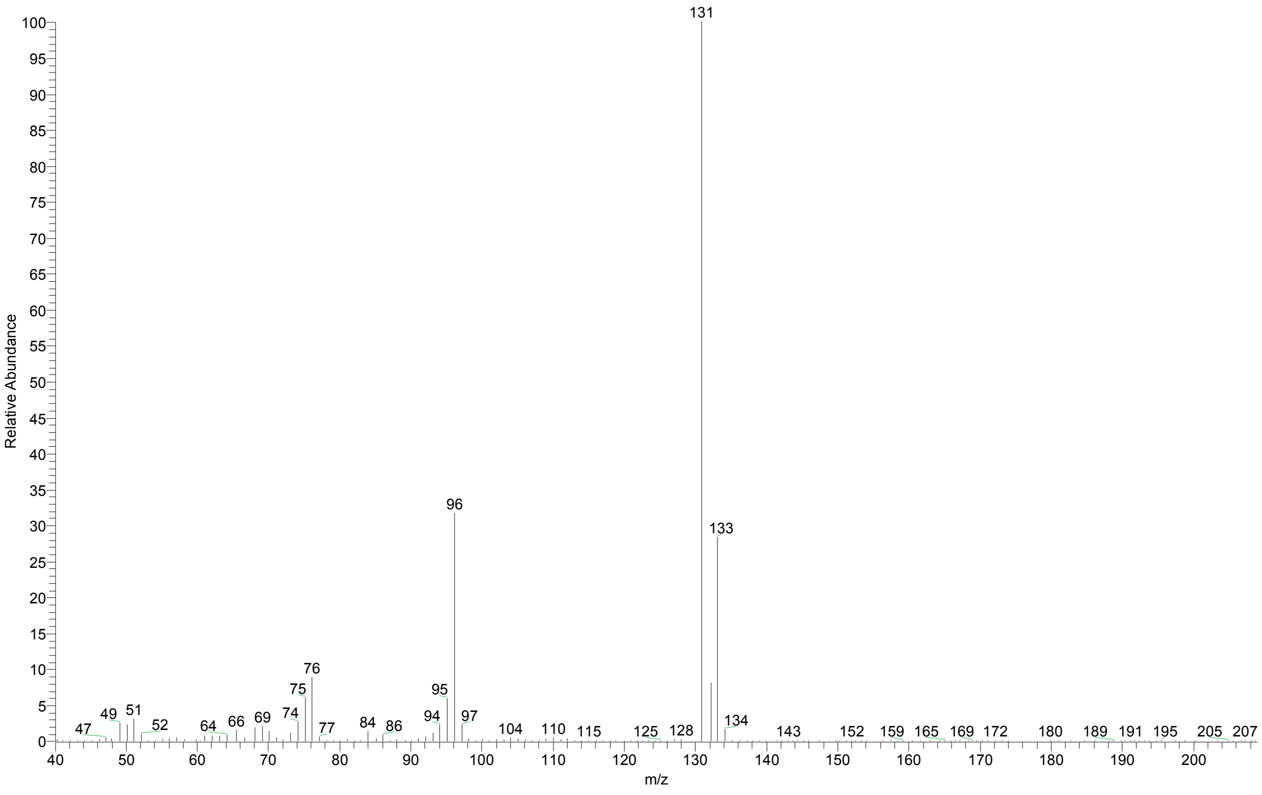

131
133
96
76
75
95
97
74
69
66
64
52
51
49
47
77
84
86
94
104
110
115
125
128
134
143
152
159
165
169
172
180
189
191
195
205
207
Relative Abundance
m/z

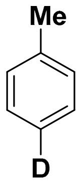

10-*d*[1]

Reference: S13

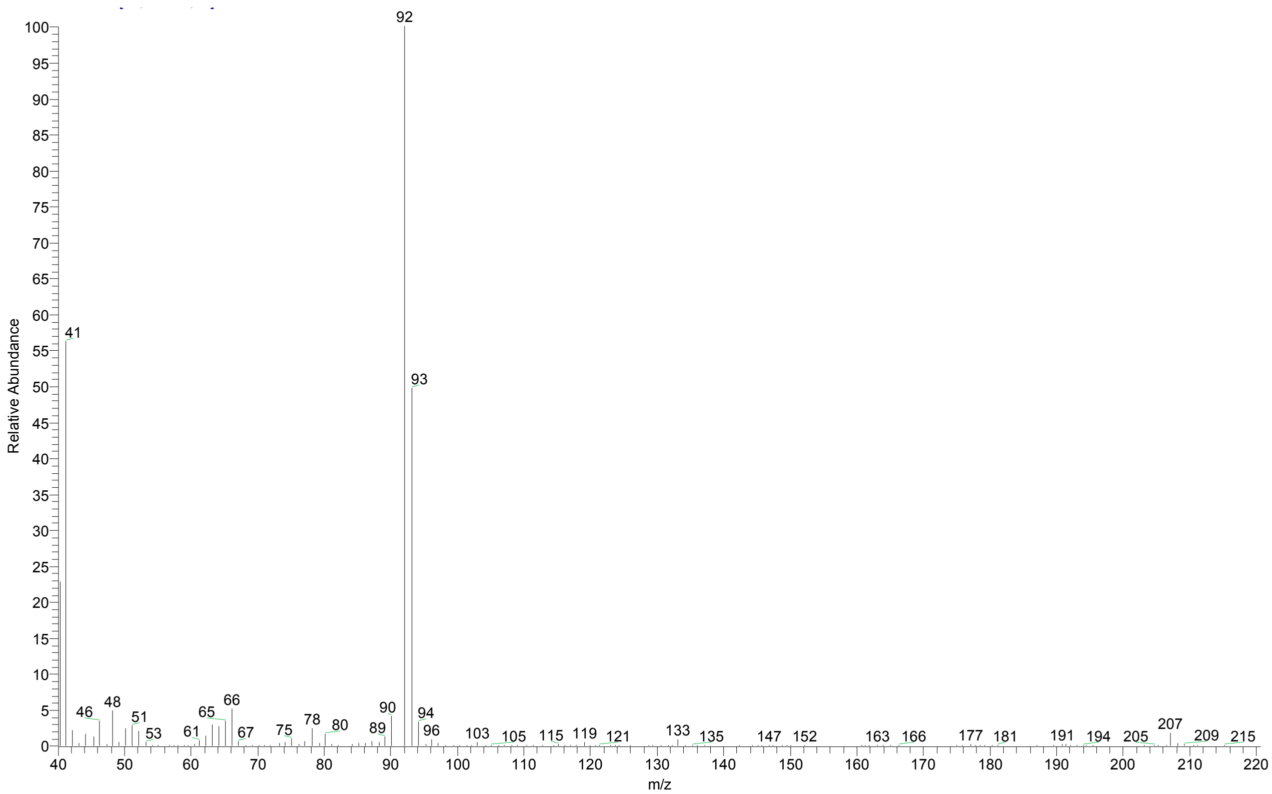

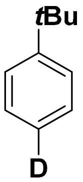

11-*d*[1]

Reference: S14

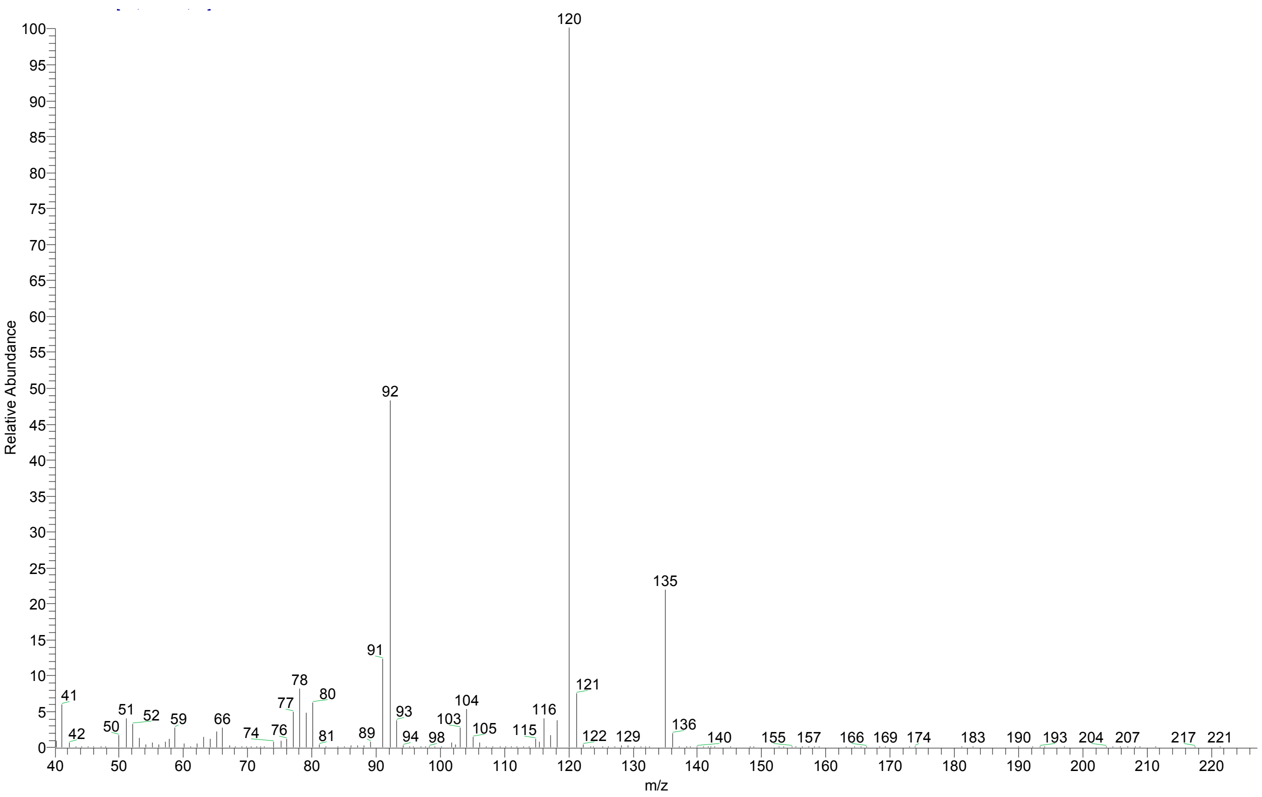

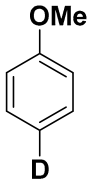

12-d[1]

Reference: S9

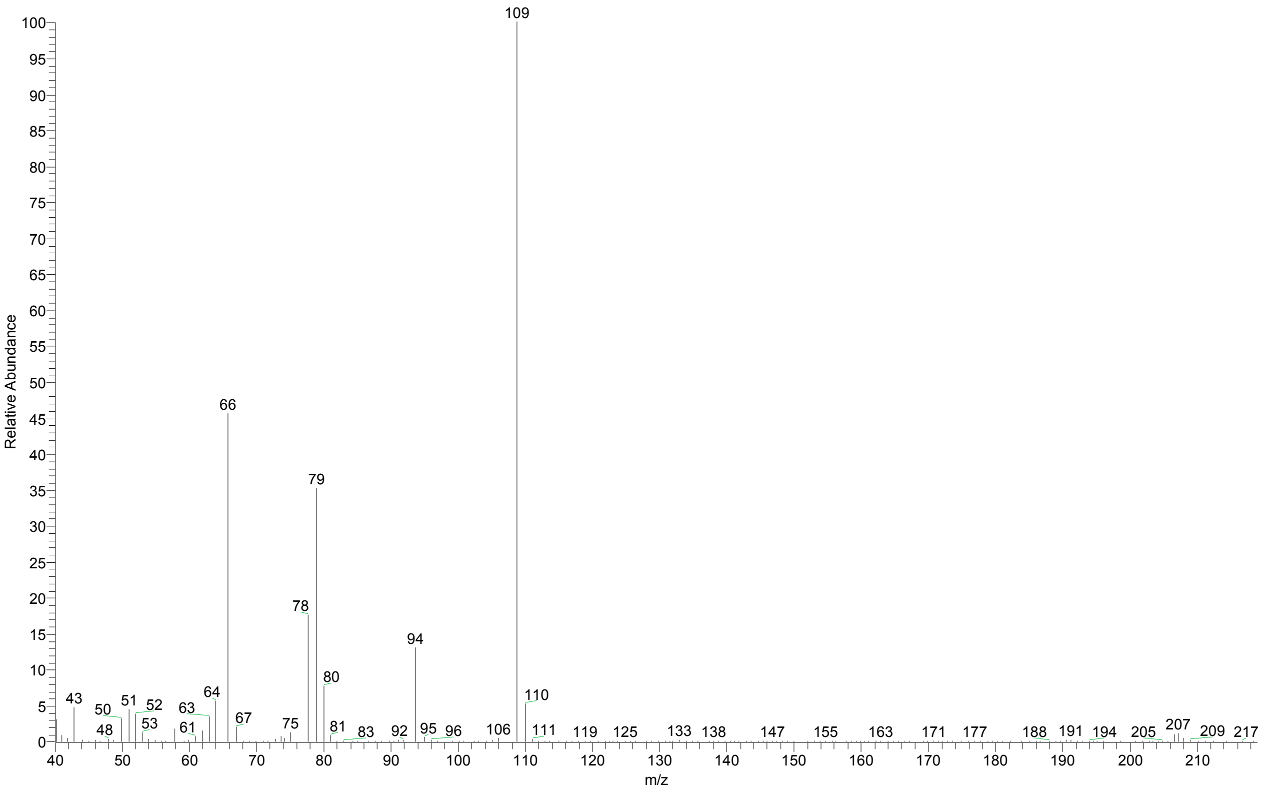

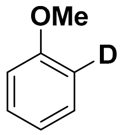

***12′-d*[1]**

Reference: S14

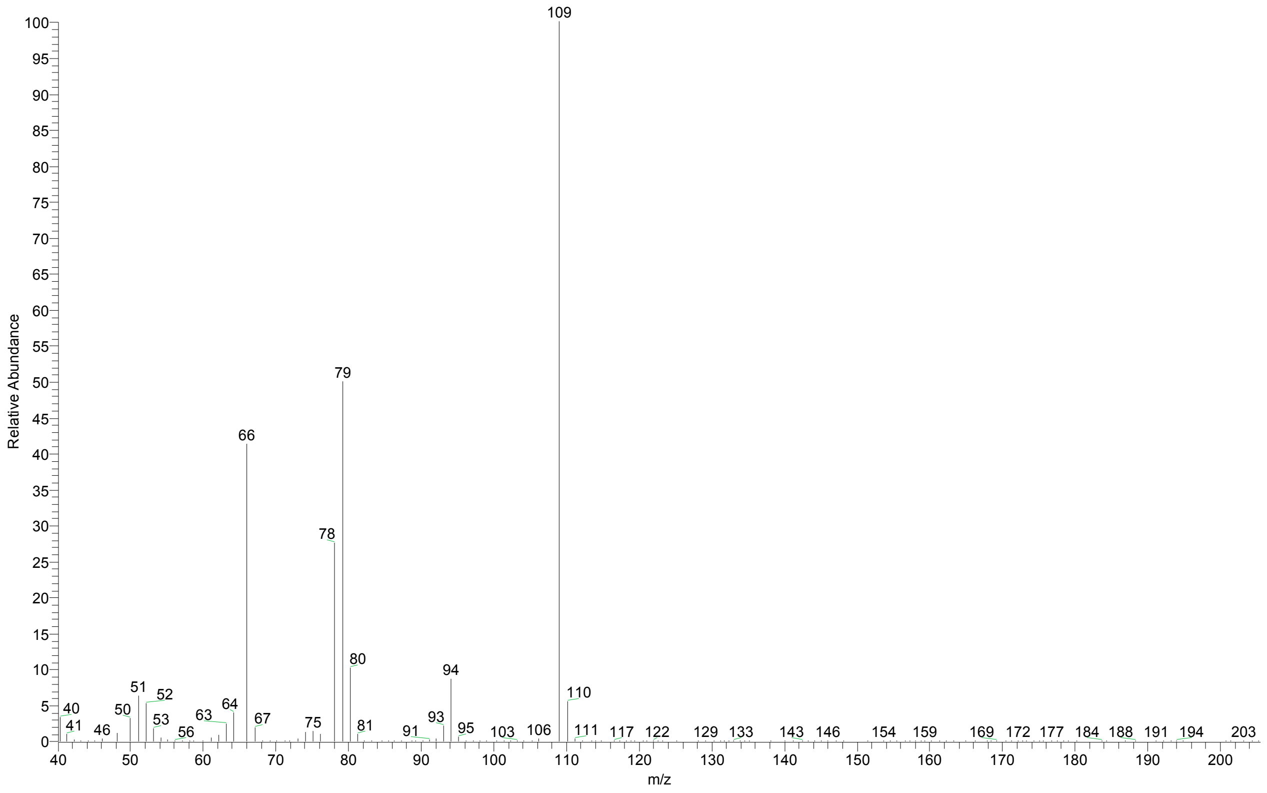

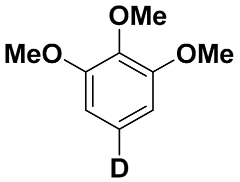

13-*d*[1]

Reference: S9

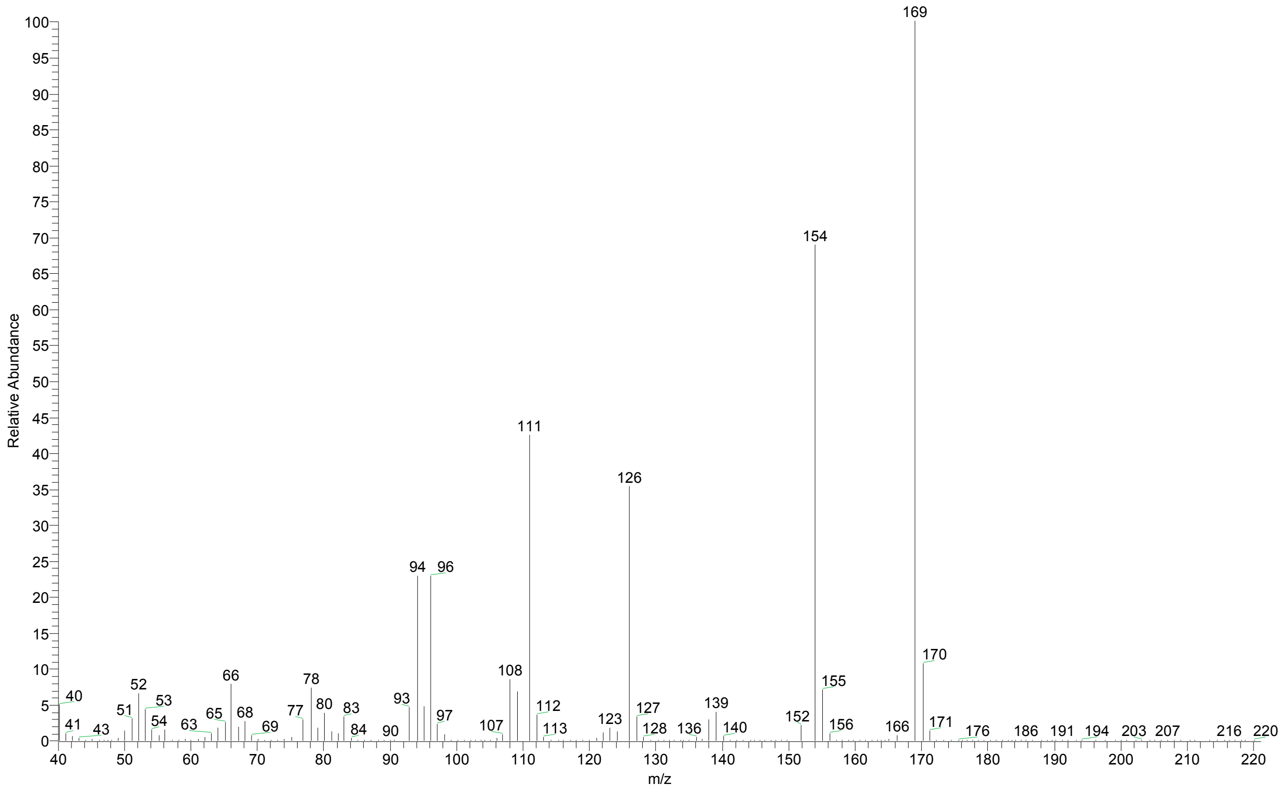

13-*d*[1] (^1H NMR, 300 MHz)

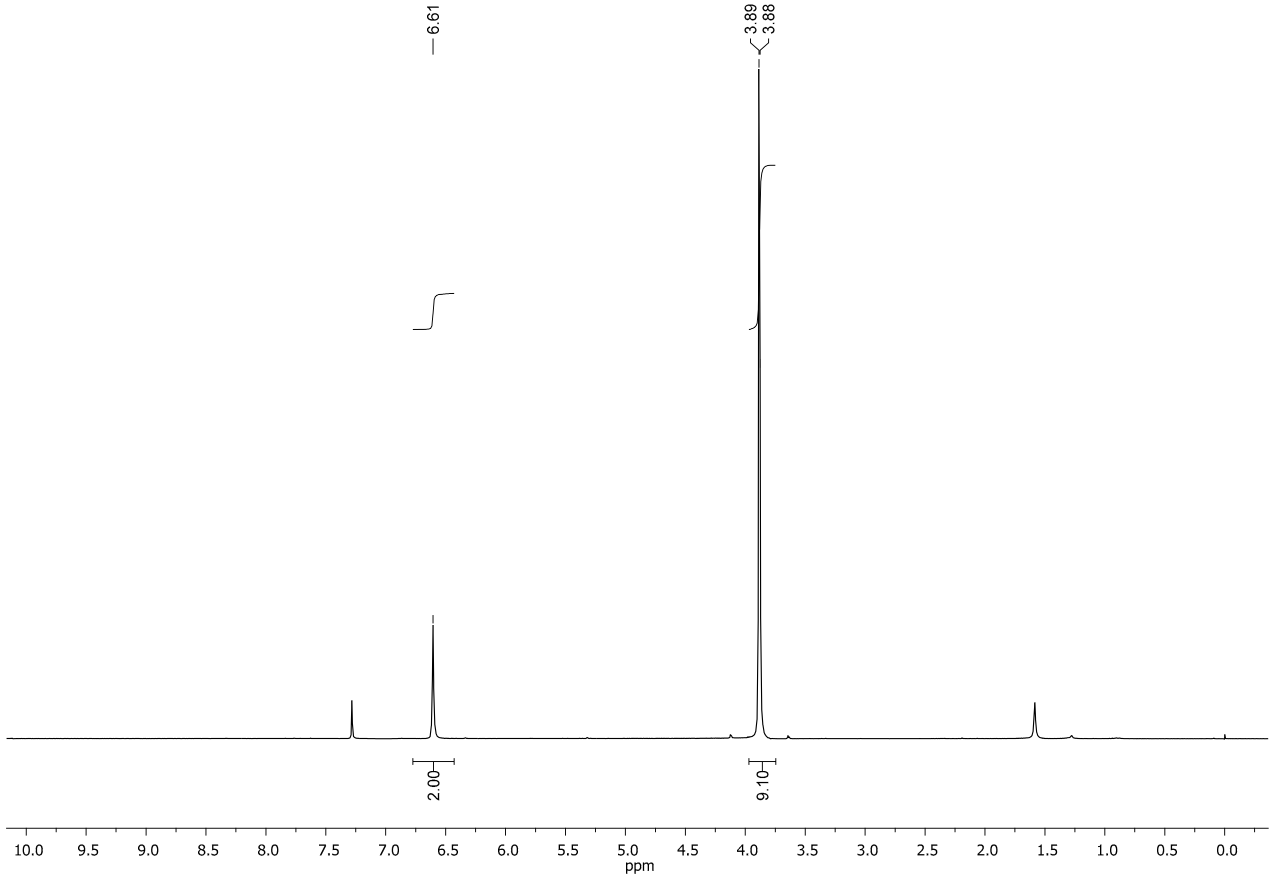

13-*d*[1] (^{13}C NMR, 75 MHz)

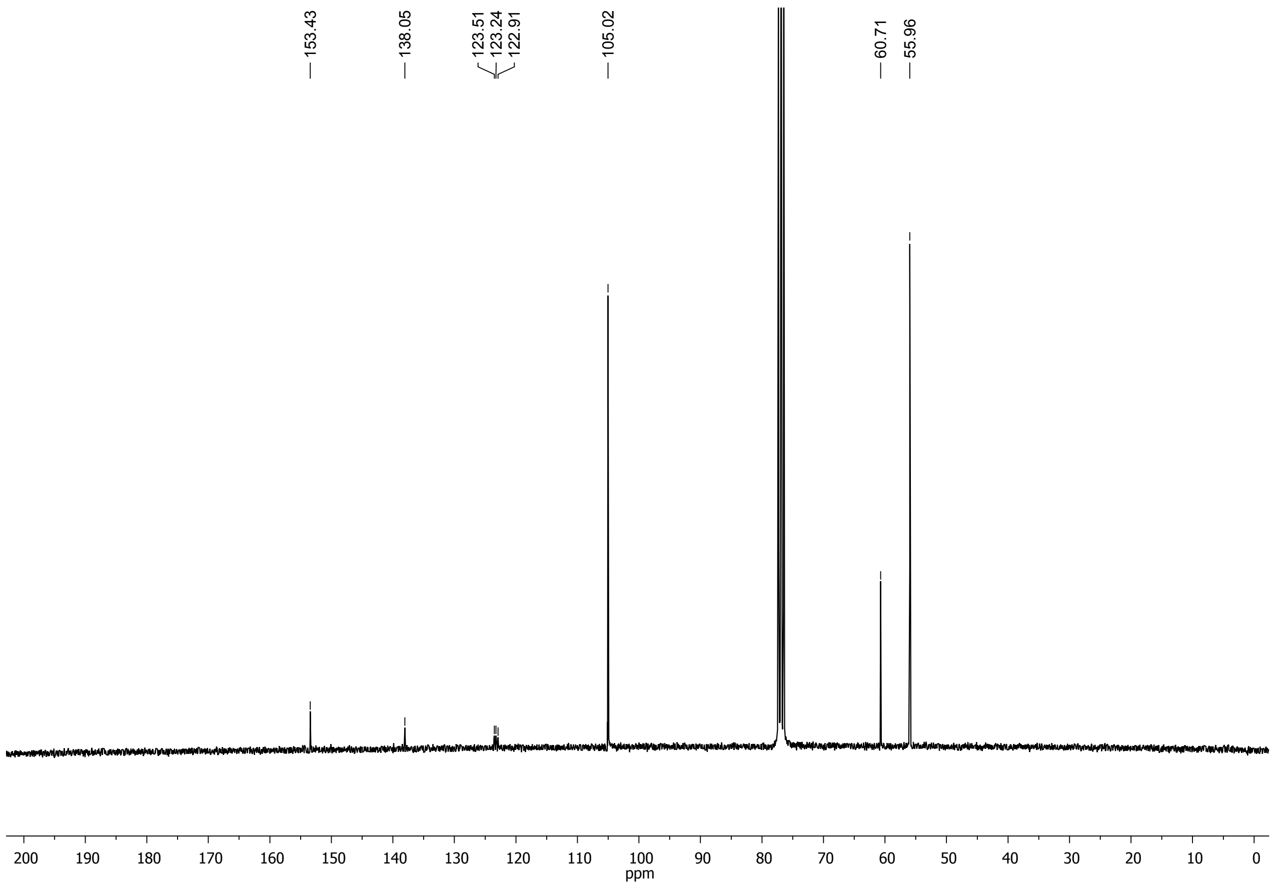

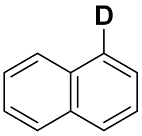

14-*d*[1]

Reference: S9

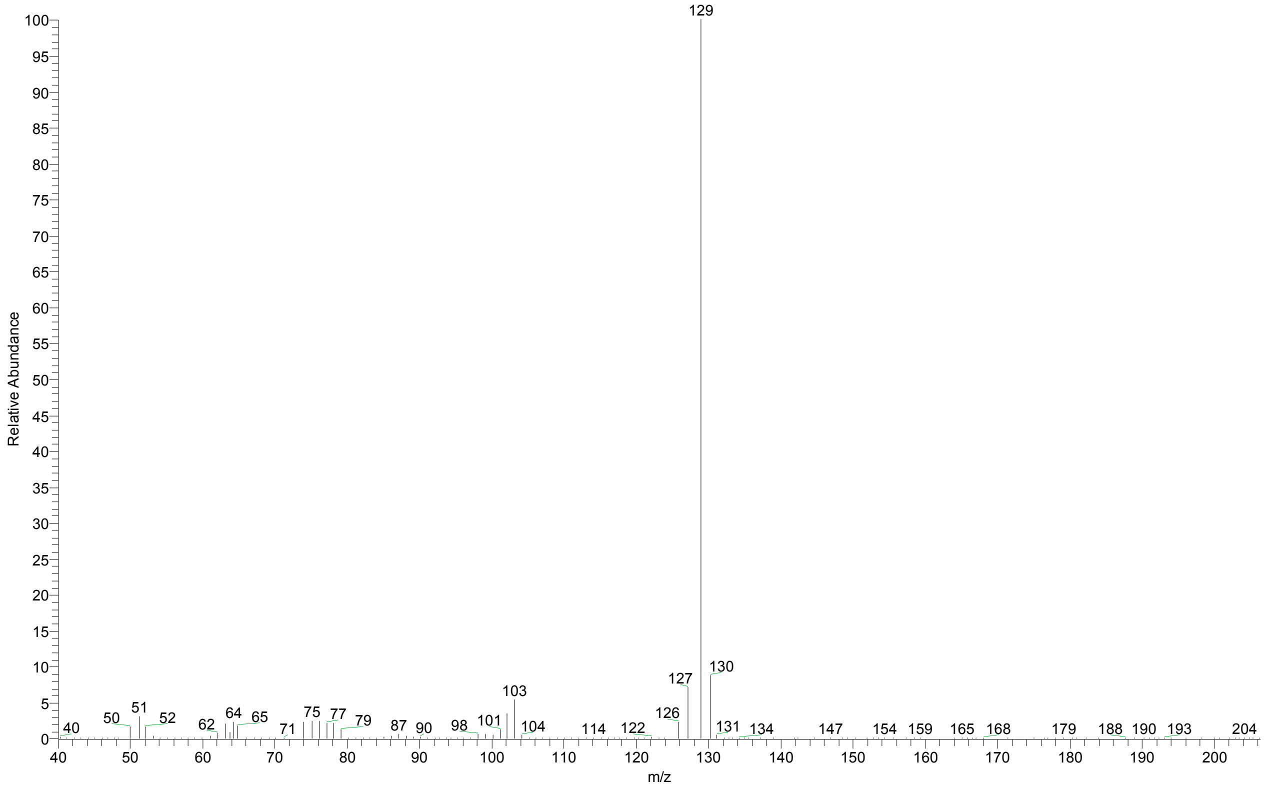

3 ^{1}H and ^{13}C NMR spectra of compounds 1h, 1i, 1k, 1l, 1q

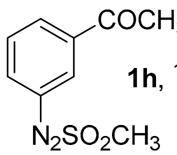

1h, ^{1}HNMR (CD_3COCD_3, 300 MHz)

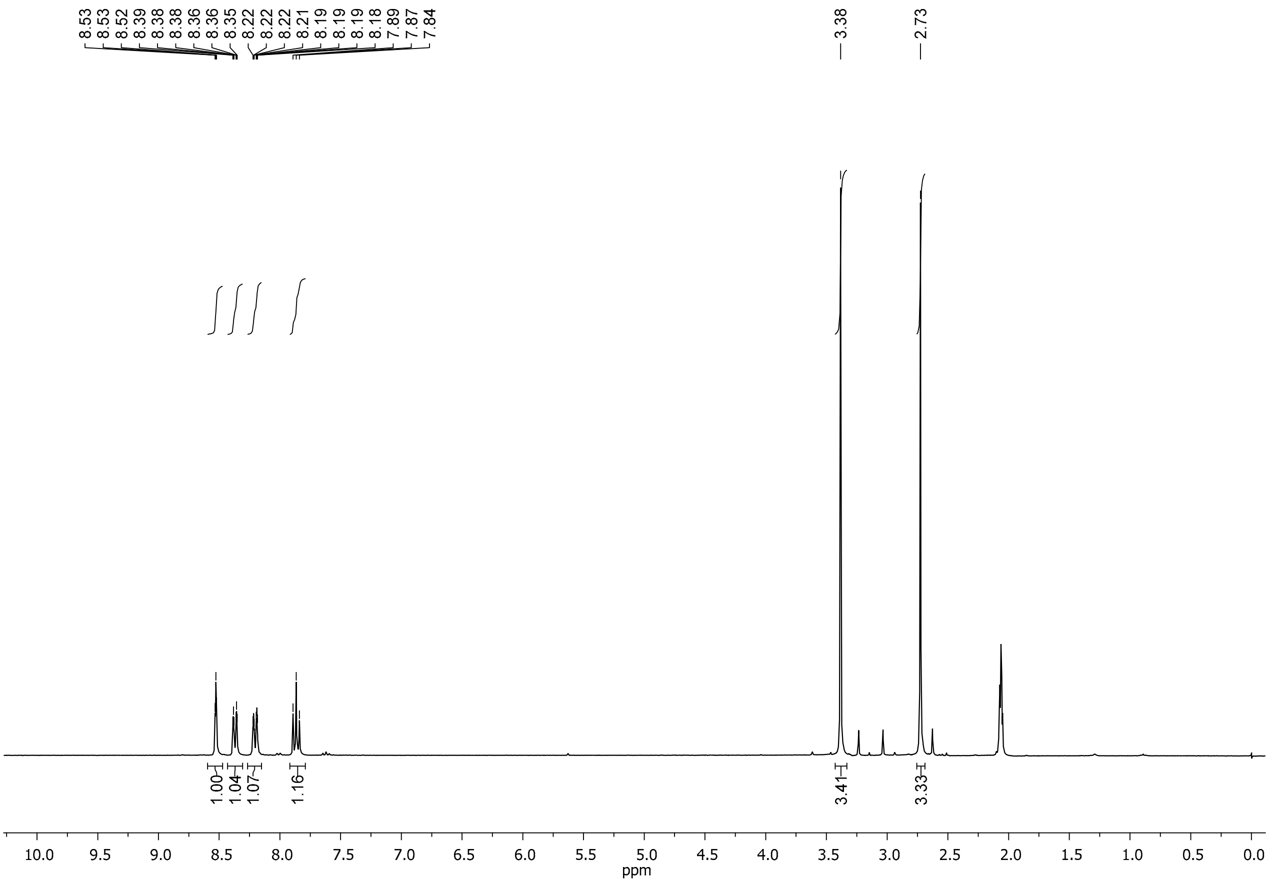

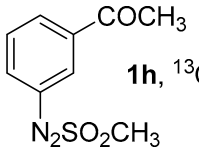

1h, ^{13}CNMR (CD_3COCD_3, 75 MHz)

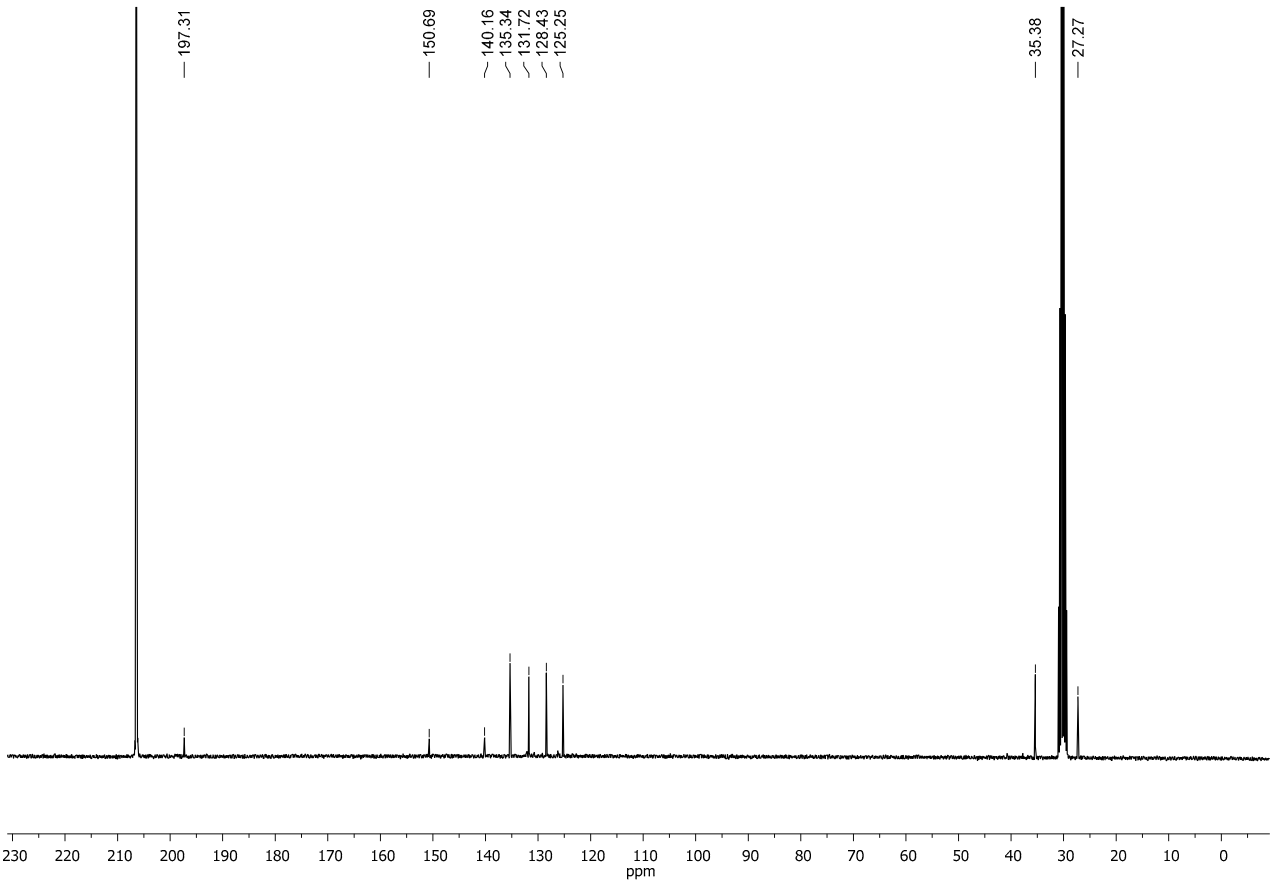

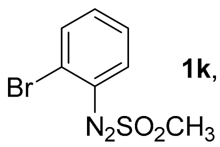

1k, ^{1}HNMR (CD_3COCD_3, 300 MHz)

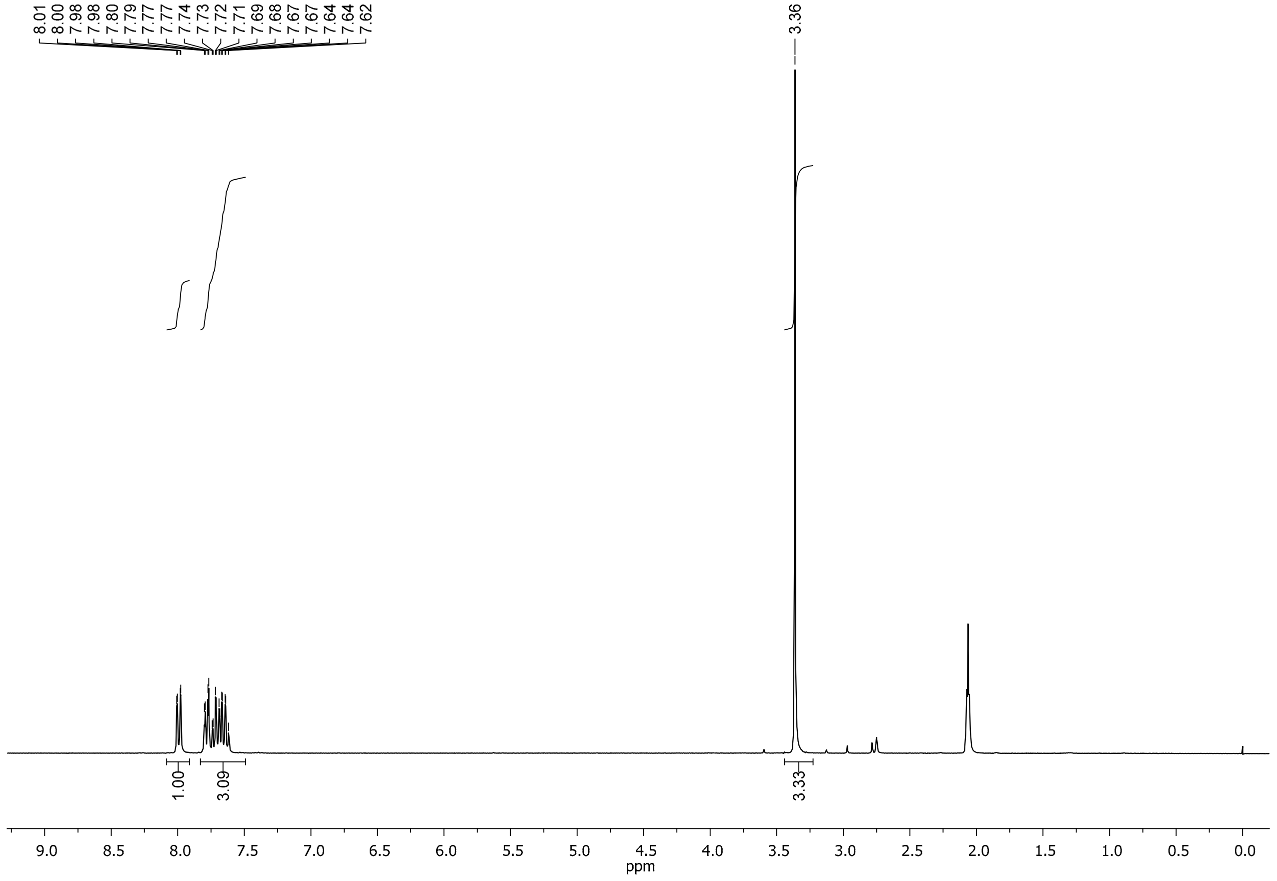

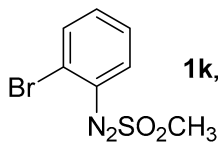

1k, ^{13}CNMR (CD_3COCD_3, 75 MHz)

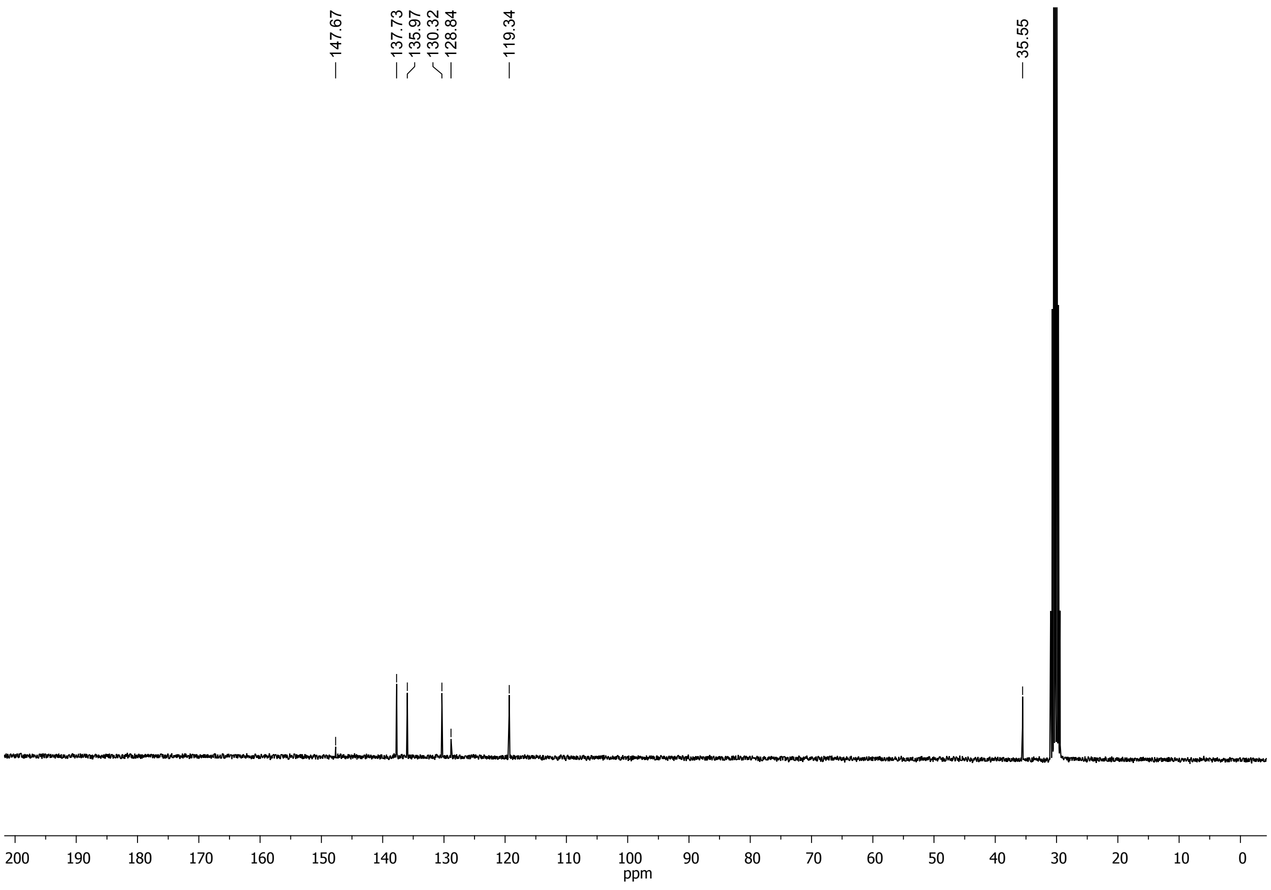

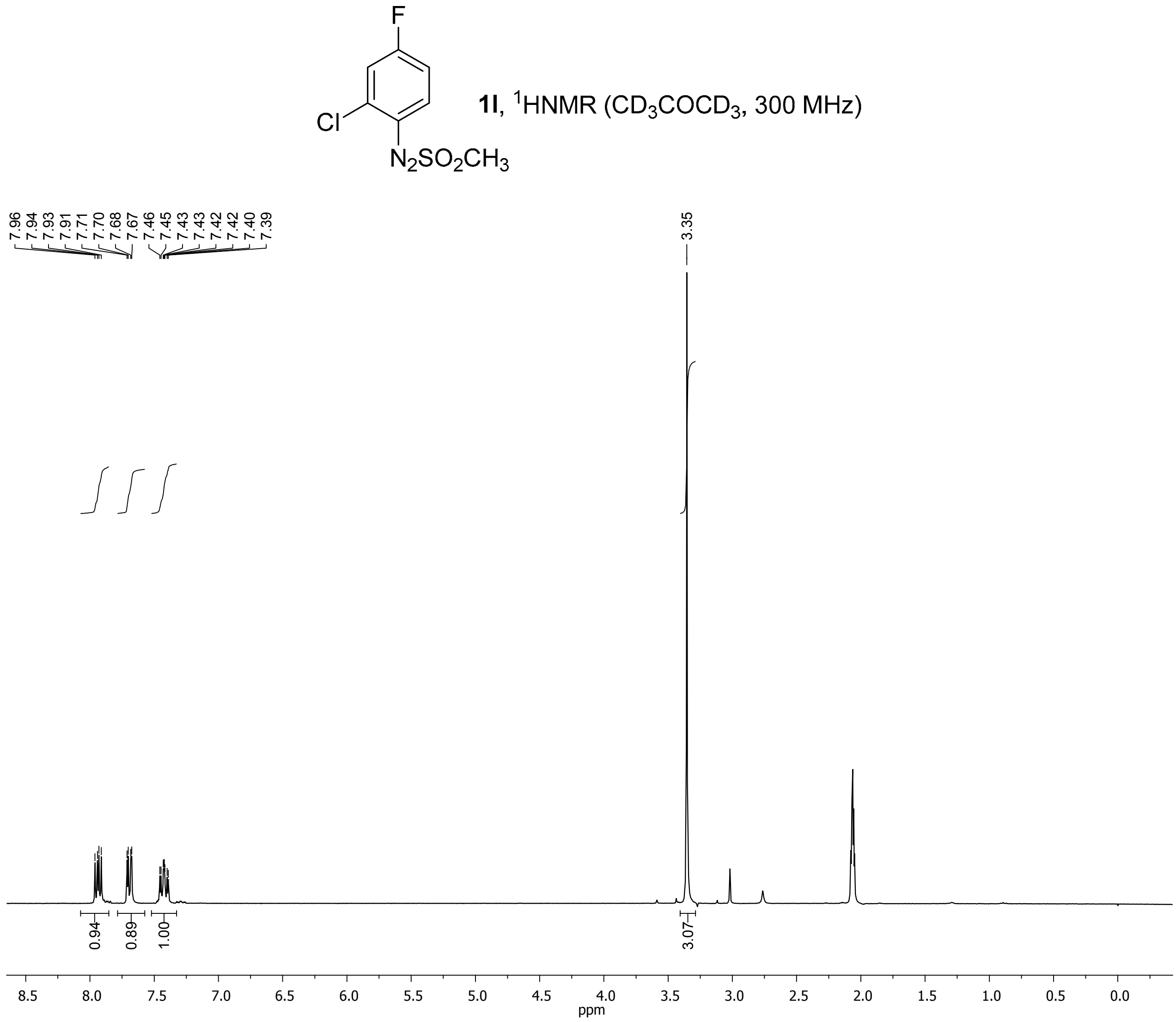

1l, ^{1}HNMR (CD_3COCD_3, 300 MHz)

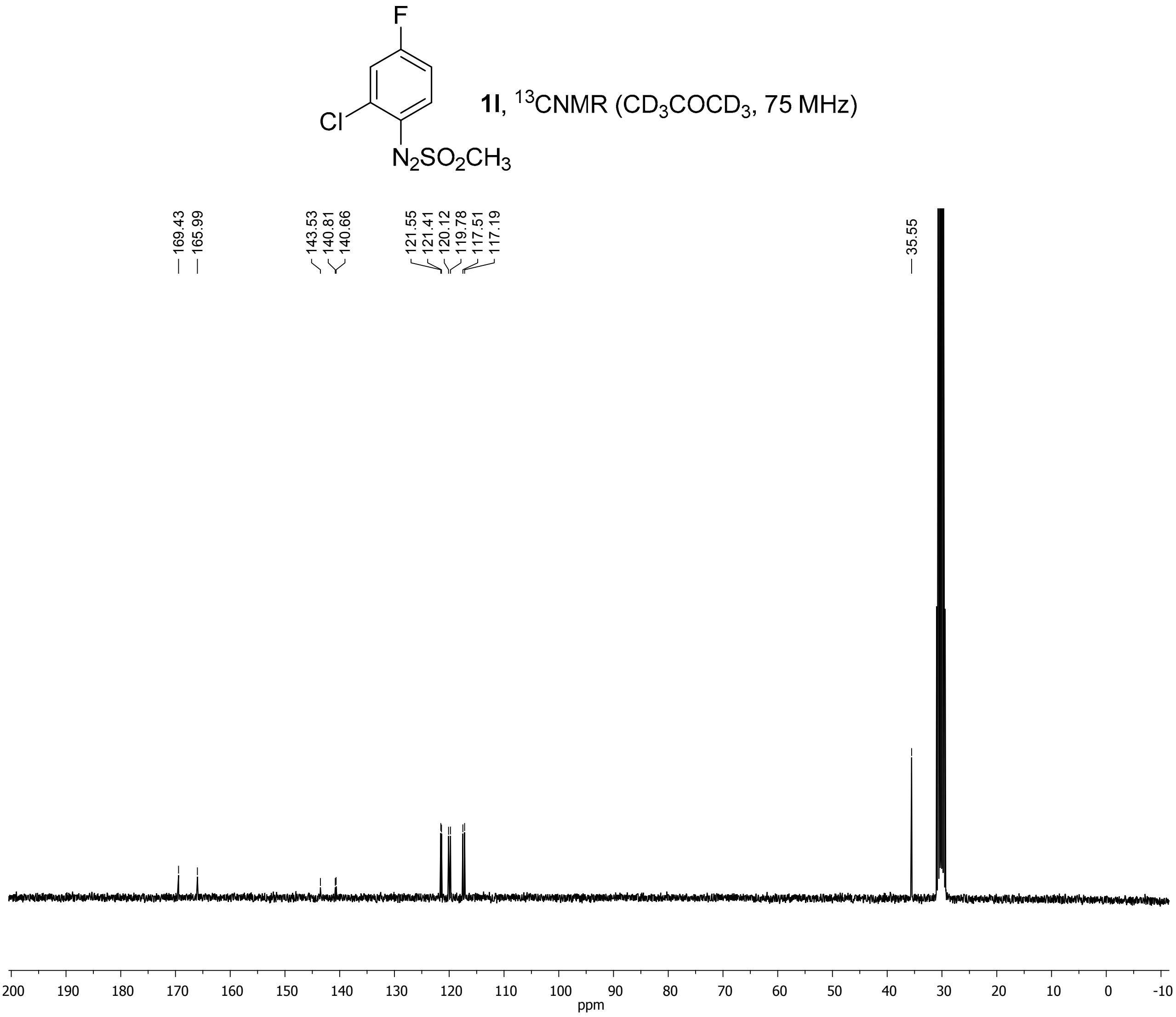

1l, ^{13}CNMR (CD_3COCD_3, 75 MHz)
169.43
165.99
143.53
140.81
140.66
121.55
121.41
120.12
119.78
117.51
117.19
35.55
ppm

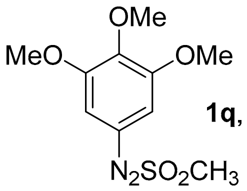

1q, ^{1}HNMR (CD_3COCD_3, 300 MHz)

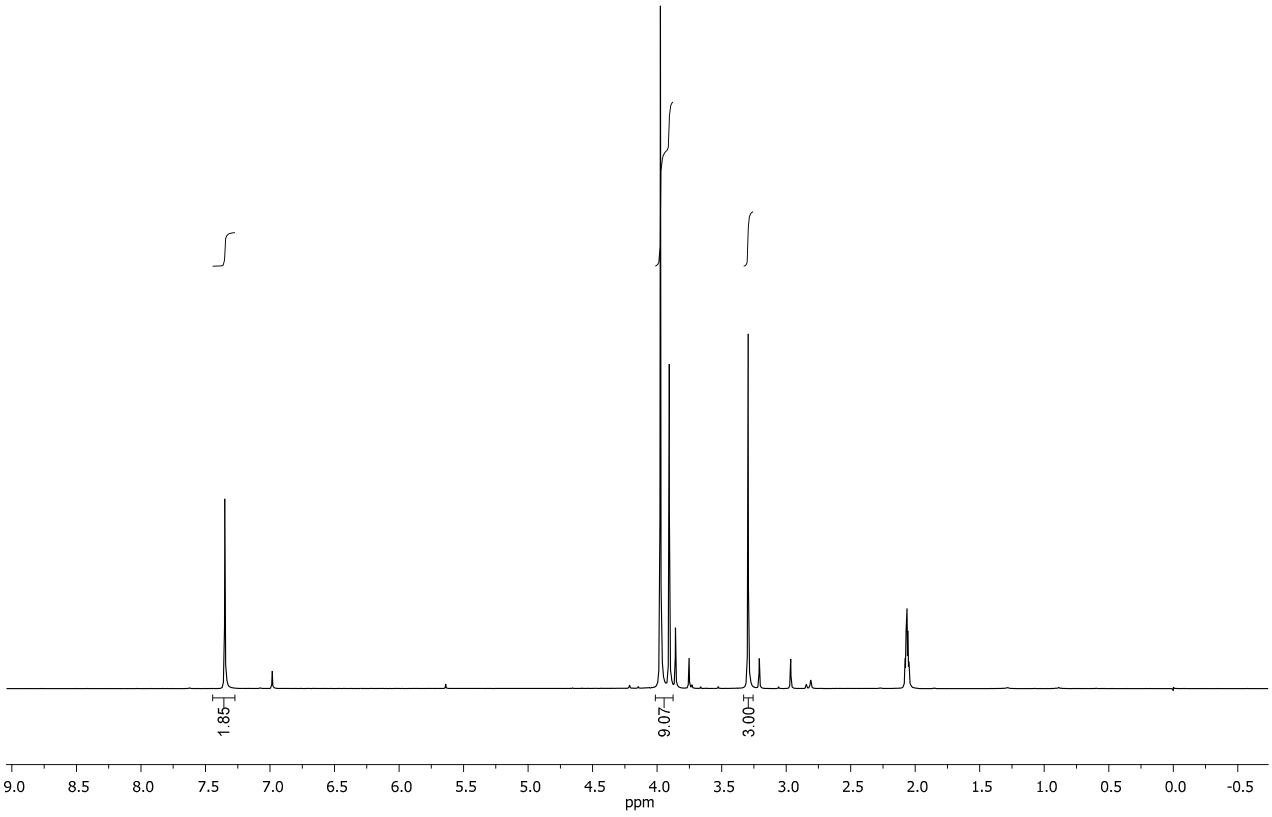

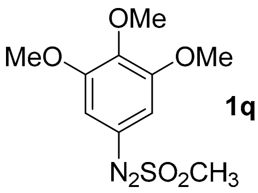

1q, ^{13}CNMR (CD_3COCD_3, 75 MHz)

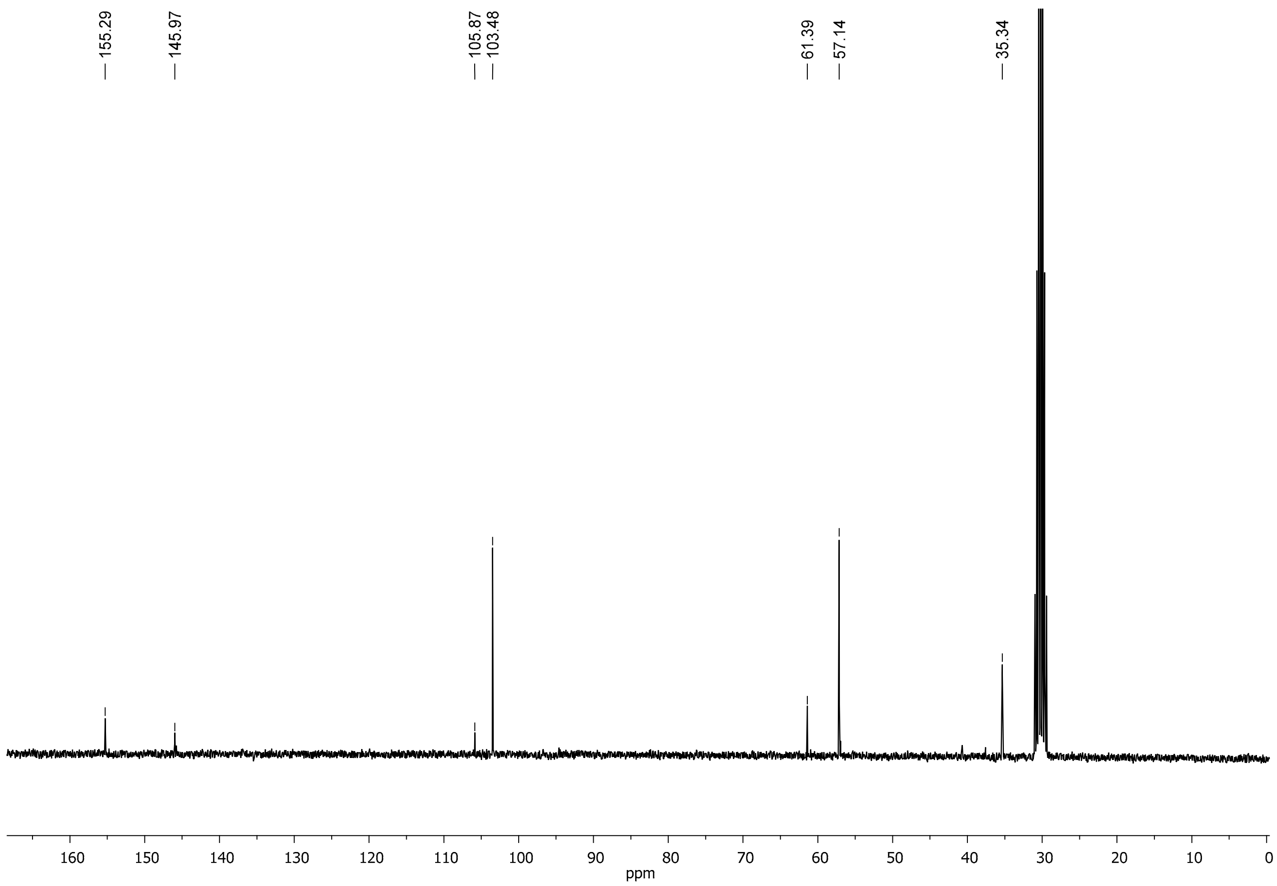

4. References

S1 Hanson, P.; Hendrickx, R. A. A. J.; Smith, J. R. L. An investigation by means of correlation analysis into the mechanisms of oxidation of aryl methyl sulfides and sulfoxides by dimethyldioxirane in various solvents, *Org. Biomol. Chem.* **2008**, *6*, 745–761. DOI: 10.1039/B714707D.

S2 Burglova, K.; Okorochenkov, S.; Hlavac, J. Efficient Route to Deuterated Aromatics by the Deamination of Anilines, *Org. Lett.* **2016**, *18*, 3342−3345. DOI: 10.1021/acs.orglett.6b01438.

S3 Janni, M.; Peruncheralathan, S. Catalytic selective deuteration of halo(hetero)arenes, *Org. Biomol. Chem.* **2016**, *14*, 3091-3097. DOI: 10.1039/C6OB00193A.

S4 Miura, Y.; Oka, H.; Yamano, E.; Morita, M. Convenient Deuteration of Bromo Aromatic Compounds by Reductive Debromination with Sodium Amalgam in CH_3OD, *J. Org. Chem.* **1997**, *62*, 1188–1190. DOI: 10.1021/jo9619037.

S5 Bank, S.; Schepartz, A.; Giammatteo, P.; Zubieta, J. Substituent effect on the electrochemical oxidation of arylmethyl anions. 3. Effect of methyl substitution on diarylmethyl anions, *J. Org. Chem.* **1983**, *48*, 3458–3464. DOI: 10.1021/jo00168a017.

S6 Berger, S; Diehl, B. W. K. Correlation between deuterium isotope effects and ^{13}C-NMR chemical shifts in substituted benzenes, *Tetrahedron Lett.* **1987**, *28*, 1243–1246. DOI: 10.1016/S0040-4039(00)95336-X.

S7 Discekici, E. H.; Treat, N. J.; Poelma, S. O.; Mattson, K. M.; Hudson, Z. M.; Luo, Y.; Hawker, C. J.; de Alaniz, J. R. A highly reducing metal-free photoredox catalyst: design and application in radical dehalogenations, *Chem. Commun.* **2015**, *51*, 11705–11708. DOI: 10.1039/C5CC04677G.

S8 Barthez, J. M.; Filikov, A. V.; Frederiksen, L. B.; Huguet, M.-L.; Jones, J. R. Lu, S.-Y. Microwave-enhanced metal- and acid-catalysed hydrogen isotope exchange reactions, *Can. J. Chem.* **1998**, *76*, 726–728. DOI: 10.1139/v98-045.

S9 Mutsumi, T.; Iwata, H.; Maruhashi, K.; Monguchi, Y.; Sajiki, H. Halogen–deuterium exchange reaction mediated by tributyltin hydride using THF-d^8 as the deuterium source, *Tetrahedron* **2011**, *67*, 1158–1165. DOI: 10.1016/j.tet.2010.12.007.

S10 Grainger, R.; Nikmal, A.; Cornella, J.; Larrosa, I. Selective deuteration of (hetero)aromatic compounds via deutero-decarboxylation of carboxylic acids, *Org. Biomol. Chem.* **2012**, *10*, 3172–3174. DOI: 10.1039/c2ob25157d.

S11 Nose, M.; Suzuki, H. Convenient One-pot Procedure for Converting Aryl Sulfides to Nitroaryl Sulfones, *Synthesis,* **2002**, *8*, 1065–1071. DOI: 10.1055/s-2002-31950.

S12 Corrie, T. J. A.; Ball, L. T.; Russell, C. A.; Lloyd-Jones G: C. Au-Catalyzed Biaryl Coupling To Generate 5- to 9-Membered Rings: Turnover-Limiting Reductive Elimination versus π-Complexation, *J. Am. Chem. Soc.* **2017**, *139*, 245−254. DOI: 10.1021/jacs.6b10018.
S13 Wang, Y.; Shen, J.; Chen, Q.; L.; Wang, He, M. Nickel-catalysed CO bond reduction of 2,4,6-triaryloxy-1,3,5-triazines in 2-methyltetrahydrofuran, *Chin. Chem. Lett.* **2019**, *30*, 409–412. DOI: 10.1016/j.cclet.2018.09.009.
S14 Liu, C.; Chen, Z.; Su, C.; Zhao, X.; Gao, Q.; Ning, G.-H.; Zhu, H.; Tang, W.; Leng, K.; Fu, W.; Tian, B.; Peng, X.; Li, J.; Xu, Q.-H.; Zhou, W.; Loh, K. P. Controllable deuteration of halogenated compounds by photocatalytic D_2O splitting. *Nat. Commun.* **2018**, *9*, 80. DOI: 10.1038/s41467-017-02551-8.
